# Human umbilical cord derived mesenchymal stem cells overexpressing HO‐1 attenuate neural injury and enhance functional recovery by inhibiting inflammation in stroke mice

**DOI:** 10.1111/cns.14412

**Published:** 2023-08-17

**Authors:** Yu Yang, Qianqian Liu, Song Deng, Qian Shao, Long Peng, Yuejuan Ling, Yue Huang, Siqi Zheng, Qiaoji Jiang, Dekang Nie, Jian Chen

**Affiliations:** ^1^ Department of Neurosurgery Affiliated Hospital of Nantong University, Medical School of Nantong University Nantong China; ^2^ Research Center of Clinical Medicine Affiliated Hospital of Nantong University Nantong China; ^3^ Department of Neurosurgery, The Yancheng Clinical College of Xuzhou Medical University The First People's Hospital of Yancheng Yancheng China

**Keywords:** brain‐derived neurotrophic factor, cytokines, heme oxygenase‐1, mesenchymal stem cells, microglia, stroke

## Abstract

**Aims:**

The current evidence demonstrates that mesenchymal stem cells (MSCs) hold therapeutic potential for ischemic stroke. However, it remains unclear how changes in the secretion of MSC cytokines following the overexpression of heme oxygenase‐1 (HO‐1) impact excessive inflammatory activation in a mouse ischemic stroke model. This study investigated this aspect and provided further insights.

**Methods:**

The middle cerebral artery occlusion (MCAO) mouse model was established, and subsequent injections of MSC, MSC^HO‐1^, or PBS solutions of equal volume were administered via the mice's tail vein. Histopathological analysis was conducted on Days 3 and 28 post‐MCAO to observe morphological changes in brain slices. mRNA expression levels of various factors, including IL‐1β, IL‐6, IL‐17, TNF‐α, IL‐1Ra, IL‐4, IL‐10, TGF‐β, were quantified. The effects of MSC^HO‐1^ treatment on neurons, microglia, and astrocytes were observed using immunofluorescence after transplantation. The polarization direction of macrophages/microglia was also detected using flow cytometry.

**Results:**

The results showed that the expression of anti‐inflammatory factors in the MSC^HO‐1^ group increased while that of pro‐inflammatory factors decreased. Small animal fluorescence studies and immunofluorescence assays showed that the homing function of MSCs^HO‐1^ was unaffected, leading to a substantial accumulation of MSCs^HO‐1^ in the cerebral ischemic region within 24 h. Neurons were less damaged, activation and proliferation of microglia were reduced, and polarization of microglia to the M2 type increased after MSC^HO‐1^ transplantation. Furthermore, after transplantation of MSCs^HO‐1^, the mortality of mice decreased, and motor function improved significantly.

**Conclusion:**

The findings indicate that MSCs overexpressing HO‐1 exhibited significant therapeutic effects against hyper‐inflammatory injury after stroke in mice, ultimately promoting recovery after ischemic stroke.

## Graphical abstract

Transfected human umbilical cord mesenchymal stem cells (hUC‐MSCs) transplanted into MCAO mice migrate to injured areas in the brain. hUC‐MSCs that overexpress HO‐1 can release high levels of brain‐derived neurotrophic factor and promote the production of M2‐type microglia. This reduces inflammatory damage after ischemia–reperfusion.

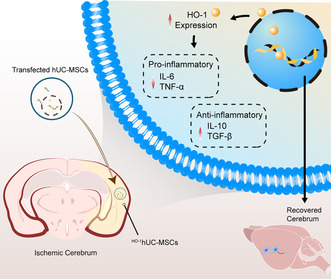

## 1 INTRODUCTION

Stroke is the second leading cause of death and the third leading cause of death and disability worldwide.^1^, ^2^ Treating cerebral ischemia is particularly challenging because of the rapid progression of the injury.^3^ Inflammatory reactions initiated at the blood–microvessel interface a few hours after the onset of ischemia underlie the transition from ischemic to inflammatory injury.^4^ A phenomenon called ischemia–reperfusion injury is often observed once blood flow is restored. This phenomenon leads to a series of pathological changes, including oxidative stress, neuroinflammation, and, ultimately, cell death through necrosis and apoptosis.^5^ ROS can be generated in the central nervous system (CNS) in various ways, mainly by microglia/astrocytes, and ROS can activate microglia to secrete pro‐inflammatory factors (TNF‐α, TGF‐β, IL‐1, and IL‐6) and exacerbate inflammation.^6^, ^7^ ROS and inflammatory factors damage microglia and astrocytes and damage adjacent cells, eventually leading to the apoptosis of neuronal cells.^8^ However, these immune cells exert neuroprotective effects against ischemia. For example, T lymphocytes may be involved in later repair processes, and microglia can remove cellular debris, promote tissue remodeling, and exert various neuroprotective effects under certain conditions.^9^, ^10^ Among these, inflammation induced by ischemia remains a key factor in secondary brain injury, and prompt anti‐inflammatory and immune adjustment strategies can effectively manage ischemia‐induced pathological treatment. Therefore, from the perspective of etiological treatment, clearing or reducing ROS and inflammatory factors after cerebral ischemia has great potential for clinical application.^11^, ^12^

Mesenchymal stem cells (MSCs) have been obtained from the placenta, including umbilical cord vessels, Wharton's jelly, amniotic/chorionic membrane, and chorionic villi. Previous studies have shown that MSC transplantation can improve neurological function recovery in animal models of CNS injuries, including middle cerebral artery occlusion (MCAO), traumatic brain injury (TBI), and spinal cord injury.^13^ Studies in recent years have demonstrated that MSCs have immunomodulatory effects on immune cells such as B and T lymphocytes, natural killer cells, dendritic cells (DCs), monocytes, and macrophages. Transplanted MSCs display immunosuppressive effects on innate and humoral immunity by inhibiting microglial cells, T lymphocyte proliferation, natural killer/B‐cell activation, and DC maturation while promoting regulatory T lymphocyte development.^14^ In addition, MSCs can migrate to injured tissues, thereby inhibiting the release of pro‐inflammatory cytokines at the site of ischemic injury and promoting the survival of injured cells. For example, the therapeutic effects of MSC transplantation in cerebral ischemia, TBI, acute lung injury, and acute renal failure have been reported. In addition, transplanted stem cells can regulate inflammation and restore neurological function.^15^ When there is a certain injury in the body, the tissues at the injury site express various signaling molecules, such as chemokines, adhesion, and growth factors. Different microenvironments secrete different signaling molecules that attract MSCs to the tissues. Transplanted neural stem cells (NSCs) can regulate the innate inflammatory response by interacting with the peripheral inflammatory system during the hyperacute phase of stroke.^16^, ^17^

The therapeutic mechanisms of MSCs in the nervous system have mainly focused on direct differentiation, neurotrophic and pro‐angiogenic growth factors secretion, immune modulation, tissue remodeling, and activation of local precursors.^18^ Heme oxygenase‐1 (HO‐1) is an important antioxidant enzyme that catalyzes the decomposition and metabolism of heme into ferrous, carbon monoxide, and biliverdin.^19^ Degradation of the heme group is conducive to preventing catalytic oxidation. In contrast, reduced bilirubin has effective ROS‐scavenging activity against peroxides, peroxynitrites, hydroxyls, and superoxide free radicals.^20^ Recent research has shown that the overexpression of HO‐1 can change the function of DCs, macrophages, and regulatory T cells and induce immune regulation.^21^, ^22^ In summary, we believe that HO‐1 overexpression in MSCs may play a role in synergistic immunotherapy.^23^

**FIGURE 1:**
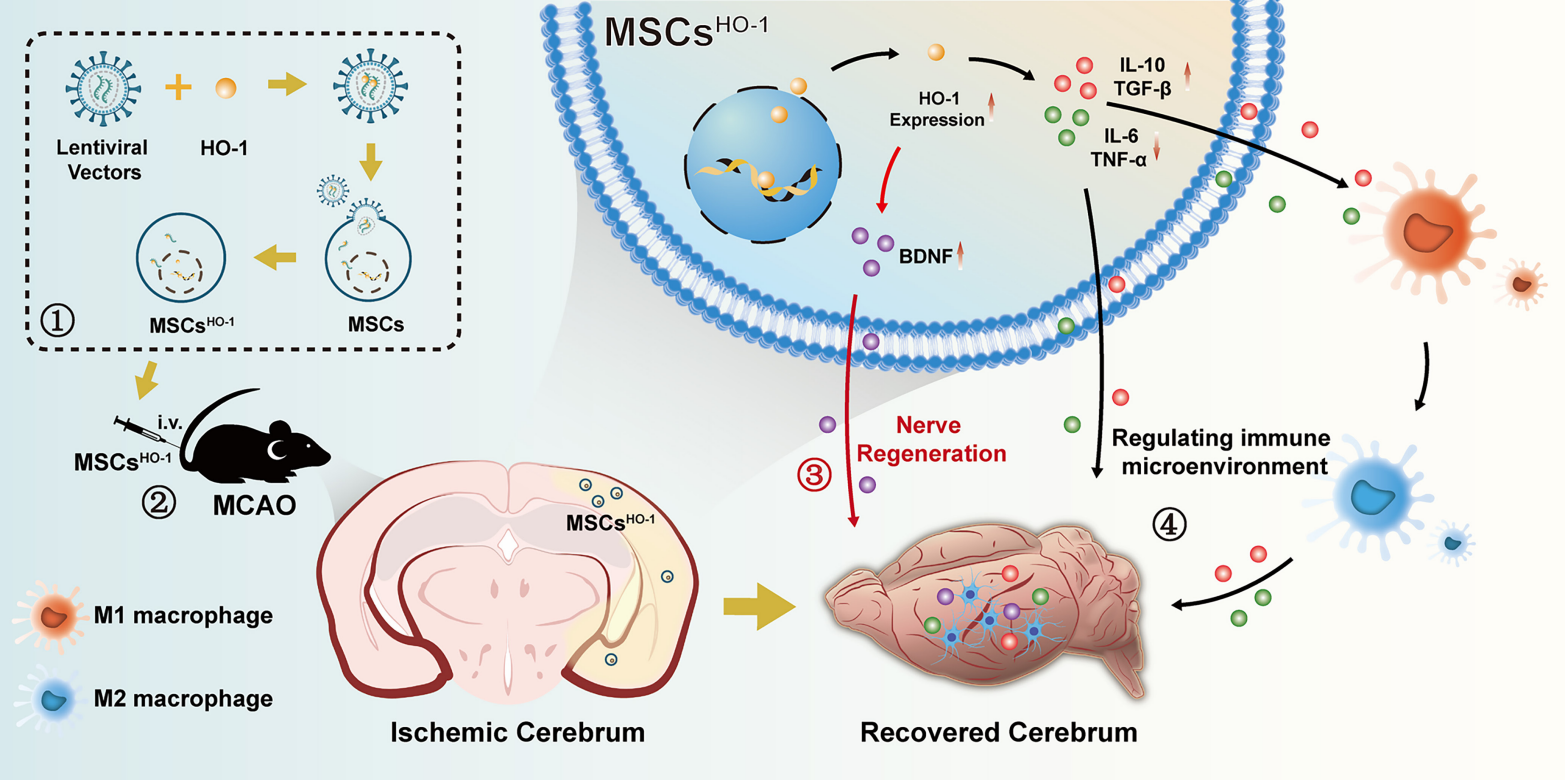
Heme oxygenase‐1 (HO‐1) transduction of human umbilical cord mesenchymal stem cells (hUC‐MSCs) for ischemic stroke treatment. ① Three‐plasmid packaged HO‐1 lentivirus‐transduced hUC‐MSCs. ② The transduced hUC‐MSCs *i.v*. transplanted to middle cerebral artery occlusion mice migrate to the injured area in the brain. ③ hUC‐MSCs overexpressed HO‐1 and released high levels of brain‐derived neurotrophic factor (BDNF). The released BDNF stimulates nerve regeneration and functional reconstruction, resulting in a significant therapeutic effect on ischemic stroke. ④ Stable expression of high levels of HO‐1 effectively reduced the expression of pro‐inflammatory factors and promoted the expression of anti‐inflammatory factors, which were released to the injury site to promote the production of M2‐type microglia and the transformation of microglia from M1 to M2. This attenuates inflammatory injury after ischemia–reperfusion.

In this study, we propose that the intravenous injection of MSCs overexpressing HO‐1 inhibits inflammation and manipulates microglial polarization to improve the efficacy of MCAO therapy. MSCs overexpressing HO‐1 reduce ROS levels effectively in the brain.^20^ More importantly, they facilitate microglial polarization into a neuroprotective M2‐like phenotype, which can secrete anti‐inflammatory cytokines and trophic factors (Figure 1).

## 2 MATERIALS AND METHODS

### 2.1 Cell culture and experimental animals

Human umbilical cord mesenchymal stem cells (hUC‐MSCs, were purchased from Cyagen Biosciences, HUXUC‐01001). Cells were placed into 75‐cm^2^ flasks (NEST Biotechnology) for expansion while observed in glass bottom cell culture dishes (NEST Biotechnology) in DMEM/F12 (1:1, v/v) containing 10% fetal bovine serum (FBS; Gibco). Cells were incubated at 37°C in a 95% air and 5% CO_2_ humidified atmosphere. Culture, amplification, cryopreservation, and resuscitation were carried out according to the recommended scheme for related cells. In this study, clean C57BL/6 mice (specific pathogen‐free level) were provided by the Experimental Animal Center of Nantong University. Five mice in a cage can form a stable social structure. All animal programs were approved by the Animal Care and Experimental Committee of Nantong University.

### 2.2 Lentivirus‐mediated HO‐1 gene insertion into hUC‐MSCs

(1) Three‐plasmid system (PxpaX2, PMD2.g, and PLVX‐HO‐1) packaging lentivirus: plasmid DNA transformation and extraction: (a) One hundred microliters of competent cells were thawed on ice and then mixed with 1 μL of plasmid DNA. (b) After incubation in an ice bath for 10 min, the centrifuge tube was placed at 42°C for 90 s (the centrifuge tube was not shaken) and then quickly placed in an ice bath for 2 min. (c) Then, 800 μL of LB liquid medium without antibiotics was added to the centrifuge tube and shaken at 37°C for 1 h (150 r min^−1^). (d) Two hundred microliters of bacterial liquid were spread on resistant LB solid medium, inverted Petri dishes, and cultured in 37°C constant temperature incubator for 12–16 h until colonies appeared. (e) One colony was taken and inoculated into the liquid medium, and cultured with shaking at 37°C for 12–16 h. (f) According to the instructions of the plasmid extraction kit, 100 μL of PxpAx2, PMD2.g, and PLVX‐HO‐1 plasmid solutions were extracted from the above bacterial liquid, and the concentrations were recorded for later use. (2) Plasmid transfection: (a) 293T cells were cultured in 6 cm and 10 cm petri dishes in advance, and transfection was carried out when the cell fusion degree reached 80%. (b) According to the mass ratio of PxpAx2: PMD2.g: PLVX‐HO‐1 = 3:2:5, the plasmid with a total mass of 27.2 μg was added to Optim‐DMEM with a total volume of 1360 μL, and the liquid was mixed 20 times by pipetting. (c) The transfection reagent Fugene (Promega) (54.4 μL) was added, mixed well, and incubated for 10–15 min at room temperature. (d) According to the proportion of 9:25, the static mixture was dropped evenly into 6 cm and 10 cm 293T cell petri dishes containing no double antibiotic; Virus collection: 48 h after the plasmid was transfected into 293T cells, the supernatant containing virus in the 10 cm petri dish was collected into a 50 mL centrifuge tube, and 8 mL DMEM complete culture medium without double antibiotic was added. After collecting the virus supernatant from the 6 cm petri dish and passing it through a 0.45 μm filter, it was used to transfect the target cells directly, and 4 mL of medium was added. (e) The peak of virus production was 72 h after continuous transfection, and the virus supernatant was collected again in the 50 mL centrifuge tube for a total of 20 mL. A syringe and a 0.45 μm filter were used for filtering. (f) The supernatant of the virus was concentrated by a 100 kDa ultrafiltration tube and centrifuged at 4000 *g* until the volume was 1 mL. After five subpackagings, it was first frozen in liquid nitrogen and then stored at −80°C.

### 2.3 Stable expression of HO‐1 in hUC‐MSCs and characterization of hUC‐MSCs

hUC‐MSCs in the fourth passage were transduced with pLVX‐HO‐1 lentiviral vectors. Puromycin (1 μg mL^−1^) was used to screen the stable hUC‐MSC^HO‐1^ line. Upon the exposure to purinomycin, all non‐transduced MSCs died within 1–2 days. The transduction efficiency was confirmed by fluorescence microscopy (BX41; Olympus).

The fresh medium was replaced every 2–3 days. When the culture reached 80% confluence, hUC‐MSCs were digested into single cell suspension with 0.25% Trypsin Express (Gibco) and washed once in 0.01 M phosphate buffer (PBS). The cells were then immediately stained for 20–30 min in the dark with the following antibodies: fluorescein isothiocyanate (FITC)‐labeled mouse anti‐human CD45; Phycoerythrin (PE)‐labeled mouse anti‐human CD29, CD73, and CD105; Allophycocyanin (APC)‐labeled mouse anti‐human CD90; And cy5.5 labeled mouse anti‐human CD34. All antibodies are listed in File S1 and all steps were performed according to the manufacturer's instructions. The distribution of hUC‐MSCs was examined by flow cytometry on BD FACScan™ (BDIS) after washing with PBS and resuspension.

### 2.4 Differentiation of human umbilical cord mesenchymal stem cells

For osteogenic differentiation, 80%–90% confluent MSCs were digested and seeded at a cell density of 2 × 10^4^ cells cm^−2^ in six‐well plates previously coated with 0.1% gelatin, with 2 mL of DMEM/F12 (Sigma) supplemented with 10% FBS (VivaCell) added to each well. When the cell confluence reached 60%–70%, the complete medium in the wells was carefully sucked away, and 2 mL of complete osteogenic differentiation medium of MSCs was added to the six‐well plate. Fresh osteogenic differentiation complete medium was used every 3 days. After 2–4 weeks of induction, the morphological changes and growth of the optic cells were stained with Alizarin Red (Sigma‐Aldrich). For chondrogenic induction of differentiation, cells after routine digestion were counted. 3–4 × 10^5^ cells were transferred to a 15 mL centrifuge tube and centrifuged at 250 *g* for 4 min. The supernatant was aspirated off, 0.5 mL of premixed solution was added, and the precipitate obtained by the previous step of centrifugation was resuspended to wash the MSCs and centrifuged at 150 *g* for 5 min at room temperature. Complete medium was resuspended in 0.5 mL of chondrogenic MSCs. The samples were centrifuged at 150 *g* for 5 min at room temperature. The centrifuge tube cap was loosened to facilitate gas exchange, and they were placed in an incubator at 37°C with 5% CO_2_ for culture. When the cell clumping occurred, the bottom of the centrifuge tube was flicked to make the cartilage pellet detached from the bottom of the tube and suspended in the liquid. From the beginning of inoculation, the cells were replaced with fresh chondrogenic induction complete medium (Gibco) every 2–3 days, about 0.5 mL chondrogenic induction differentiation complete medium per tube. After fluid exchange, the bottom of the centrifuge tube was flicked to remove the cartilage pellet from the bottom of the tube and suspend it in the liquid. The centrifuge tube cap was loosened slightly and placed into an incubator at 37°C with 5% CO_2_ to continue the induction culture. After continued induction for 21–28 days, the cartilage spheres could be sectioned by formalin fixation and paraffin embedding, and finally stained with Alcian Blue (Gibco). All cells were imaged using light microscopy (CKX41; Olympus).

### 2.5 Animal middle cerebral artery occlusion model

Male C57BL/6 mice weighing approximately 20 g were purchased from the experimental animal center, and all mice undergoing surgery were anesthetized with isoflurane using a small animal gas anesthesia machine (ABS small animal anesthesia machine; Yuyan Instruments). Mice were subjected to transient MCAO for 90 min by the intraluminal suture technique and reproducible ischemic lesions in the unilateral striatum and cortex. Carefully, the common carotid artery (CCA), external carotid artery (ECA), and internal carotid artery (ICA) were exposed via a midline cervical incision under an operating microscope (Leica). The proximal CCA and ECA can be ligated with 6‐0 silk thread. Then, the ICA was clamped temporarily with a microsurgical clip. Then, a silicon rubber‐coated round‐tip nylon surgical thread was inserted into the ICA via a small puncture in the CCA to occlude the origin of the middle cerebral artery (MCA; Figure 4). The silk suture around the CCA was tightened to prevent bleeding from the puncture site. Ninety minutes later, the suture was withdrawn to maintain reperfusion. Body temperature was maintained at 37°C by placing the mice on a heating pad throughout the procedure.

### 2.6 In vivo cell homing analysis

hUC‐MSCs^HO‐1^ were incubated with 1,1‐dioctadecyl‐3,3,3′,3′‐tetramethylindocarbocyanine iodide (DiI, Solarbio, CAS: 41085‐99‐8) for 0.5 h at 37°C. Cells were washed twice and analyzed by flow cytometry. hUC‐MSCs^HO‐1^ (5 × 10^5^ cells) labeled with DiI were injected intravenously into five MCAO mice 1 day after MCAO. After 4, 8, 16, and 24 h of circulation, three MCAO mice at each time point were sacrificed to collect brain, lung, liver, heart, kidney, and spleen tissue. These organs were immediately studied using an in vivo imaging system (Tanon ABL X6 animal living imaging system, IVIS Image living imaging software matched with the instrument).

### 2.7 Cell transplantation and behavioral testing

The animals were randomly allocated into four groups: (i) SHAM (sham‐operated control, *n* = 8); (ii) MCAO (MCAO‐operated mice treated with physiological saline only, *n* = 8); (iii) MSCs (MCAO mice treated with hUC‐MSCs through the internal jugular vein, *n* = 8); and (iv) MSCs (the MCAO^HO‐1^ mice treated with hUC‐MSCs^HO‐1^ through the internal jugular vein, *n* = 8). Transplantation was performed into the intracarotid artery 24 h after MCAO. Mice were transplanted with cells 24 h after MCAO. The dose of transplanted cells was 5 × 10^5^. After cell transplantation, behavioral tests and body weight measurements were performed on Days 1, 4, 7, 14, and 28 by two investigators blinded to the experimental groups. According to previous reports (File S2), the Modified Neurological Severity Score (mNSS) test is a combination of motor, sensory, reflex, and balance tests used to assess neurological function. The normal score is 0, and the maximum defect score is 18. A higher score indicates a more severe injury. At the same time, the falling time of the mice was detected with an accelerating rotarod. Mice were trained daily to walk on an accelerating rotarod (Ugo Basile) [5 (10 s)‐20 (10 s)‐30 r.p.m. acceleration in 5 min] for 6 days. Each mouse was tested three times a day with a 5‐min interval between trials. The trial ended when the mouse fell off the rod. For this analysis, we calculated the average latency for each mouse to fall from the 3 trials performed daily.

### 2.8 Triphenyltetrazolium chloride staining

After the treatment, after the mice (three each in the MCAO, hUC‐MSC, hUC‐MSC^HO‐1^, and SHAM groups) were anesthetized, the brains were removed by breaking the neck, and the brains were placed in the brain trough. Place in the refrigerator at −80°C for 1–2 min, and freeze the brains slightly to harden. The brains were sectioned coronally into 2‐mm‐thick sections. The brain slices were placed in 2% triphenyltetrazolium chloride (TTC) solution and placed in a 37°C incubator in the dark for 15 min. The brain slices were shaken halfway to make the staining uniform. The stained brain slices were placed in 4% formalin solution, fixed, and stored for subsequent quantitative detection of the infarct area.

### 2.9 Western blotting

Western blot analysis was performed to detect HO‐1 expression in hUC‐MSCs and hUC‐MSCs^HO‐1^. To do this, the cells in the dish were scraped and centrifuged, and the cell pellet was resuspended and incubated with 300 μL of RIPA lysis buffer (Solarbio) and 3 μL of PMSF for 30 min on ice. The suspension was centrifuged at 12,000 *g* for 30 min at 4°C. Protein concentrations were measured with a biospectrophotometer (Thermo Fisher Scientific, NanoDrop™ One/OneC Microvolume UV–Vis Spectrophotometer, ND‐ONE‐W, U.S.A.). Approximately 30 μg of protein was separated by 10% sodium dodecyl sulfate–polyacrylamide gel electrophoresis (SDS–PAGE) and then transferred to a polyvinylidene fluoride (PVDF) membrane. Membranes were blocked and incubated with primary antibodies against β‐actin (Sigma, A3854) and HO‐1 (ab68477), followed by anti‐rabbit secondary antibodies (Beyotime, A0208). Protein bands were visualized with enhanced chemiluminescent substrate (ECL) by using a chemiluminescent gel imaging analyzer (Bio‐Rad Universal Hood II, 76S/06439). We used ImageJ to process and analyze the image to obtain the gray values (IntDen) of different protein bands and then normalized the target proteins. Taking the gray value of β‐actin bands as the base, the ratio between the gray value of other protein bands and that of β‐actin bands is the relative optical density, which represents the relative protein expression.

### 2.10 Enzyme‐linked immunosorbent assay (ELISA)

After the treatment, mice (three each in the MCAO, hUC‐MSC, hUC‐MSC^HO‐1^, and SHAM groups) were sacrificed 3 days after MCAO operation. The brains were removed and ground with saline with a tissue homogenizer (JXFSTPRP24, China) to produce a 10% homogenate. The concentrations of inflammatory factors and brain‐derived neurotrophic factor (BDNF) in each sample were measured by IL‐1β, IL4, IL‐6, IL‐10, IL‐17, TNF‐α, TGF‐β, and BDNF ELISA kits (File S3) according to the manufacturer's instructions.

### 2.11 Quantitative real‐time (qRT‐)PCR analysis

Levels of gene expression of eight cytokines [IL‐1Ra, IL‐1β, IL‐4, IL‐6, IL‐10, IL‐17, tumor necrosis factor (TNF)‐α, and transforming growth factor (TGF)‐β] and BDNF (File S4) were analyzed by qRT‐PCR in hUC‐MSCs and hUC‐MSCs^HO‐1^. Cells were cultured in 100 mm dishes until they reached 90% confluence. Total RNA from hUC‐MSCs or HO‐1 hUC‐MSCs was isolated with TRIzol reagent (Beyotime, R0016), and total mRNA was determined by a biospectrophotometer at a wavelength of 260 nm. cDNA was synthesized using HiScript III RT SuperMix for qPCR (+gDNA wiper) (Vazyme). The cDNA was mixed with AceQ qPCR SYBR Green Master Mix (Low ROX Premixed) (Vazyme) and the primers (forward, reverse) for IL‐1β, IL‐4, IL‐6, IL‐10, IL‐17, TNF‐ α, TGF‐β, and BDNF (File S4). The β‐actin (File S4) gene was used as a reference gene. Relative gene expression was quantified by the $2^{-\Delta \Delta C_{T}}$ method.^24^

### 2.12 Immunostaining assessment

The 10 μm thick sections were collected on poly‐L‐lysine‐coated glass slides and treated for hematoxylin–eosin (HE) staining and immunofluorescence analysis. For immunofluorescence analysis, sections were first blocked in 10% goat serum in PBST (0.01 M PB containing 0.05% v/v Tween 20) for 1 h at RT and then treated with rabbit anti‐NeuN (1:500; Novus), mouse anti‐GFAP (1:1000; Proteintech), Iba1 (1:500; Wako) and CD206 (1:1000; Proteintech) antibodies overnight at 4°C followed by incubation with Alexa Fluor™ 594‐conjugated goat anti‐rabbit IgG (Invitrogen) and Alexa Fluor™ 488‐conjugated goat anti‐mouse IgG (Invitrogen). The nuclei of the total cells were indicated by DAPI staining. Fluorescence signals were visualized under a fluorescence microscope (Zeiss Axio Imager. 2). GFAP represented astrocytes, NeuN represented neurons, Iba1 represented microglia, and CD206 represented M2‐type cells.

### 2.13 Flow cytometry

On the fifth day after MCAO, mice (three each) in the MCAO, hUC‐MSC, hUC‐MSC^HO‐1^, and SHAM groups were perfused with normal saline, and the brains were taken and immersed in phosphate buffered saline (PBS). Right brain tissue was removed with forceps and placed in DMEM containing 5% FBS, and the brain tissue was dissociated by trituration with the flat stopper of a 1 mL syringe. The cell suspension was filtered through a 40‐μm filter (BD Biosciences) to prevent cell clumping and centrifuged at 1000 *g*. Then, 37% Percoll separation solution was added and centrifuged at 1000 *g* to keep the cell pellet. Cells were washed with 1% BSA and PBS, centrifuged at 1000 *g* and incubated with CD11b (eBioscience), CD86 (Biolegend), and CD206 (eBioscience) antibodies for 30 min on ice. Data were collected using a Pentalaser BD LSRFortessa (BD Biosciences) and analyzed using FlowJo software.

### 2.14 Statistical analysis

All data were analyzed by GraphPad software (GraphPad Prism 8.0) and presented as mean ± standard deviation (SD). We used the Shapiro–Wilk test to assess the normality of the distribution of continuous variables. All data are tested for normality, and data that does not show a normal/Gaussian distribution is analyzed by nonparametric equivalents. *p* < 0.05 was considered statistically significant. Use two‐way ANOVA to analyze survival, weight, mNSS, and rotarod. Other experimental data were compared using one‐way ANOVA. Student's *t*‐test was used to analyze data if only two groups were applied. For non‐normally distributed data, the Mann–Whitney *U*‐test was applied to determine the differences between two groups, and Kruskal–Wallis one‐way ANOVA was employed to evaluate the existence of differences among the three groups, all pairwise was selected for multiple comparisons.

## 3 RESULTS

### 3.1 HO‐1 is stably overexpressed in MSCs^HO^ ^‐1^

**FIGURE 2:**
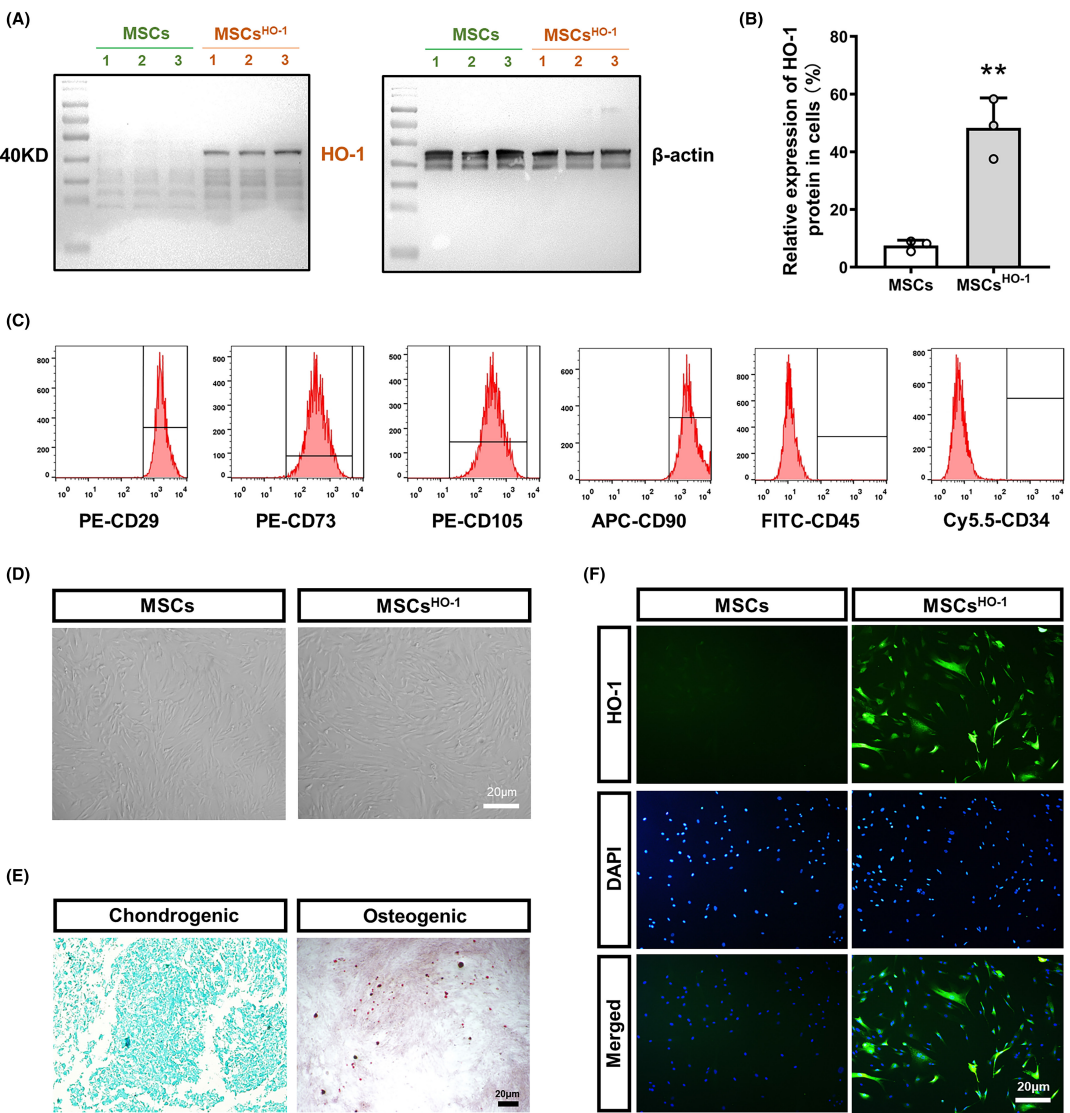
Construction and characterization of engineered mesenchymal stem cells (MSCs) overexpressing heme oxygenase‐1 (HO‐1). (A) HO‐1 (left) and Actin (β‐Actin) (right) expression in the overexpression and control groups was detected by western blotting. (B) Quantitative analysis of HO‐1/β‐Actin by Western Blot. **p < 0.01, MSCs^HO‐1^ vs. MSCs. Student's *t*‐test (*n* = 3). (C) Analysis of CD29, CD73, CD105, CD90, CD45, and CD34 expression on MSCs^HO‐1^ by flow cytometry. (D) Morphology of MSCs and MSCs^HO‐1^ under bright field (BF) microscopy. (E) Alcian blue staining after chondrogenic differentiation of MSCs^HO‐1^ (left) and alizarin red staining after osteogenic differentiation (right). Scale bar, 20 μm. (F) After infection with lentivirus, the expression of HO‐1 in the overexpression group and the control group was detected by immunofluorescence (green), and DAPI showed the localization of the nucleus. Scale bar, 20 μm.

The target gene, HO‐1, was analyzed by western blotting (Figure 2A,B, File S5) and immunocytofluorescence (Figure 2F), confirming its successful transduction. This finding indicates that the expression of HO‐1 in MSCs^HO‐1^ was significantly higher than that in control cells (MSCs). Bright‐field microscopy revealed no obvious changes in the morphology of MSCs^HO‐1^ before and after transduction (Figure 2D). MSCs and MSCs^HO‐1^ showed spindle‐forming fibroblast‐like morphology. For osteogenic differentiation (Figure 2E) and chondrogenic differentiation (Figure 2E), MSCs^HO‐1^ showed a normal ability to induce differentiation. Flow cytometry was used to identify the immunophenotype of MSCs^HO‐1^. Our results showed that hUC‐MSCs^HO‐1^ expressed stromal/MSC markers CD29, CD73, CD90, and CD105 but not hematopoietic or endothelial markers CD34 and CD45 (Figure 2C, File S6).^23^ Bright‐field micrographs, induced differentiation images, and flow‐through assays demonstrated that the phenotype and morphology of MSCs^HO‐1^ were unaffected by transduction.

### 3.2 Cytokine and BDNF levels of MSCs^HO^ ^‐1^

**FIGURE 3:**
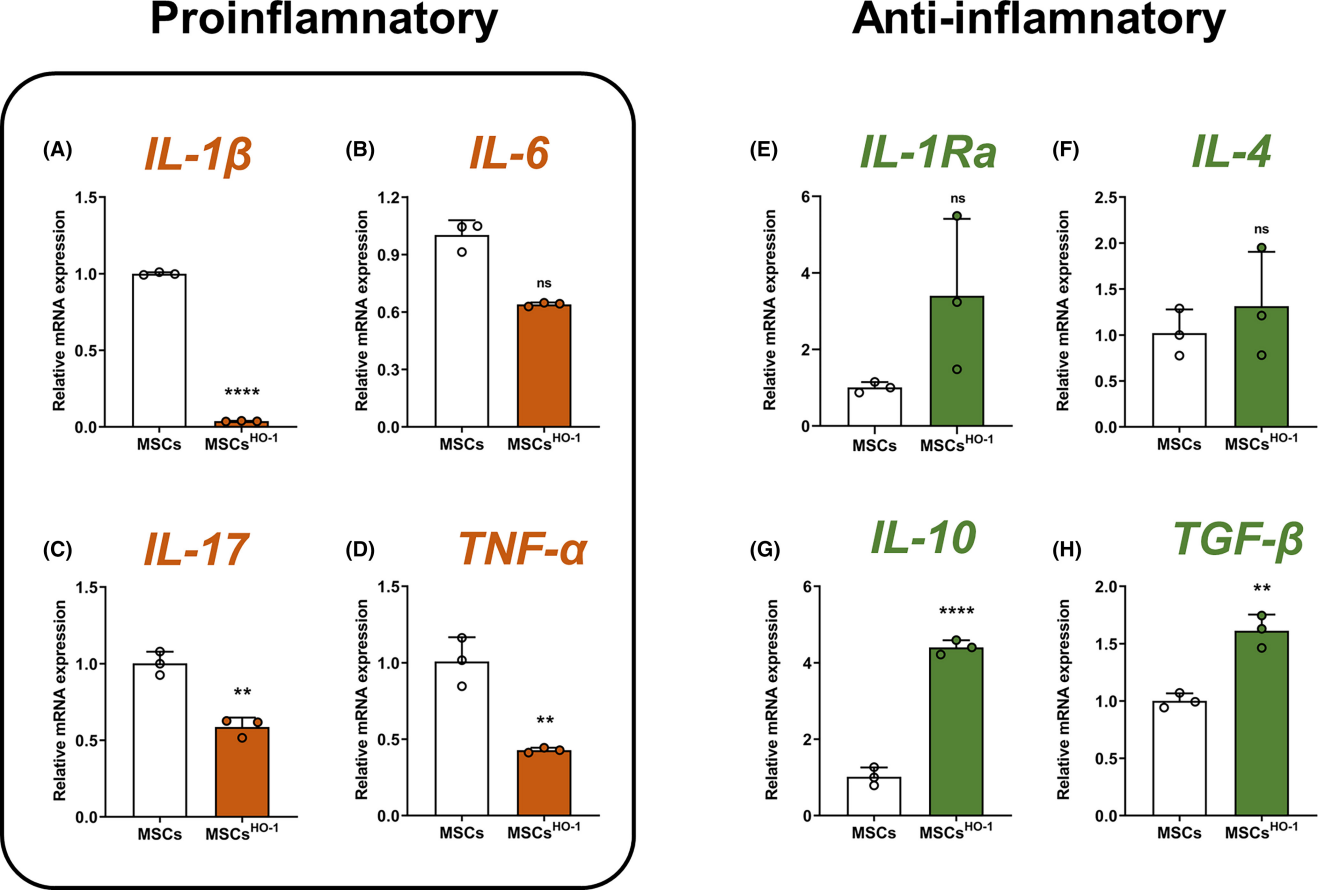
mRNA expression of factors. (A) IL‐1β, (B) IL‐6, (C) IL‐17, (D) TNF‐α, (E) IL‐1Ra, (F) IL‐4, (G) IL‐10, and (H) TGF‐β. Data on the mRNA expression levels were shown as mean ± SD. **p < 0.01 and ****p < 0.0001, mesenchymal stem cells (MSCs^HO‐1^) vs. MSCs. Student's *t*‐test (*n* = 3).

Regarding the expression of inflammatory gene markers, compared with MSCs, IL‐1β, IL‐17, and TNF‐α (Figure 3A–D) were downregulated in MSCs^HO‐1^, while IL‐1β downregulation was significant. In addition, the data of the MSCs group of IL‐6 experimental results did not pass the normality test (*p* = 0.0494 < 0.05, Shapiro–Wilk test). The Mann–Whitney *U*‐test showed no significant differences between the two groups. Therefore, we cannot conclude that IL‐6 transcription declines after HO‐1 overexpression in MSCs. TGF‐β and IL‐10 were significantly upregulated, but the upregulation of IL‐1Ra and IL‐4 was not statistically significant (Figure 3E,F). These statistical results suggest that pro‐inflammatory factors such as IL‐1β, IL‐17, and TNF‐α were decreased in MSCs^HO‐1^, while the expression of anti‐inflammatory factors such as IL‐10 and TGF‐β was increased. MSCs^HO‐1^ showed higher BDNF expression than MSCs (Figure 8E).

### 3.3 Establishment of the mouse MCAO model and distribution of transplanted MSCs in vivo

**FIGURE 4:**
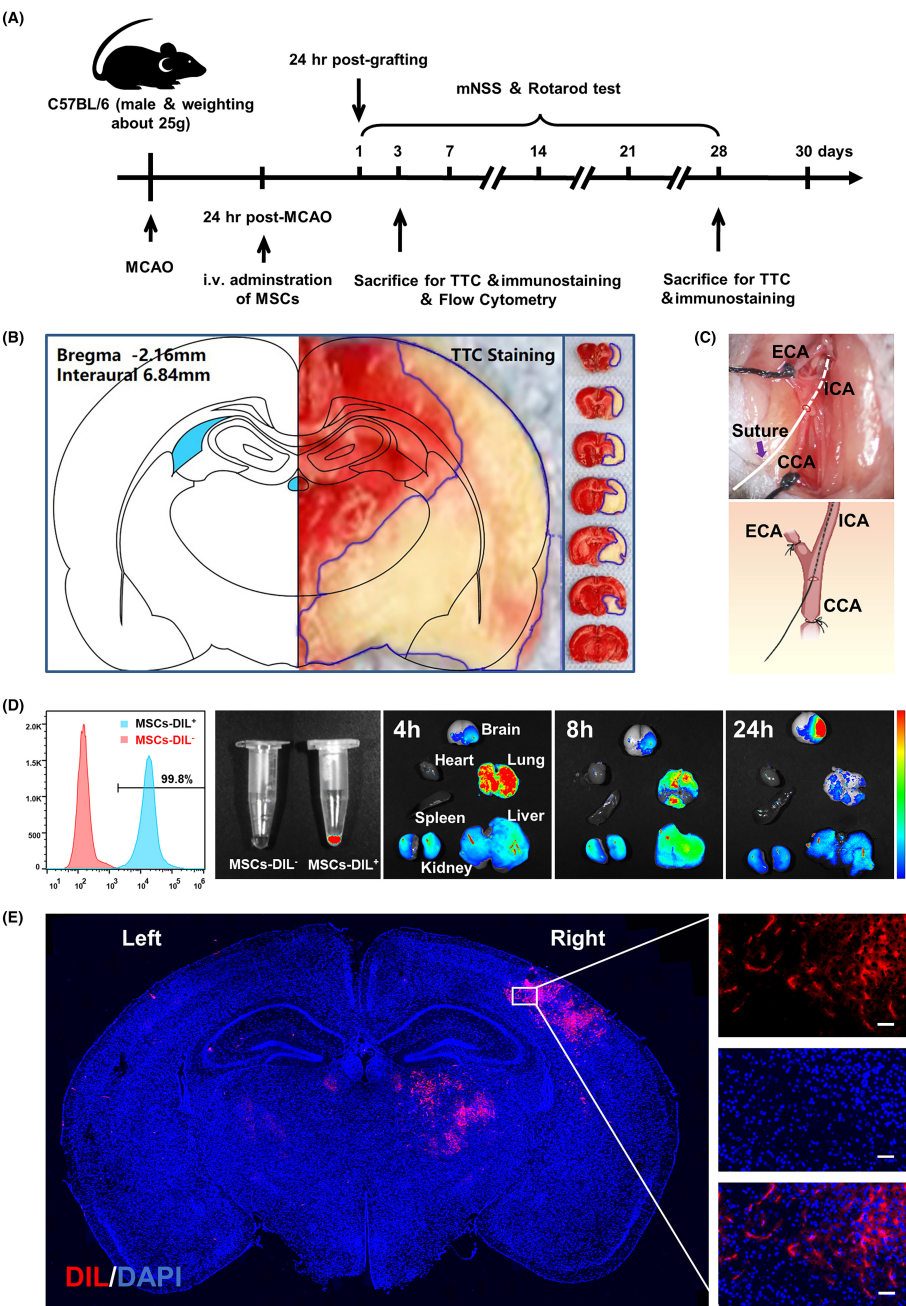
Construction of middle cerebral artery occlusion (MCAO) model mice and homing and enrichment of mesenchymal stem cells (MSCs^HO‐1^) in the brain ischemic injury site of MCAO model mice. (A) Schematic illustration of tail vein injection of MSCs and MSC^HO‐1^ treatment of MCAO model mice. (B) Schematic diagram of 2,3,5‐triphenyltetrazolium chloride staining of brain slices 24 h after mouse MCAO modeling. (C) Indwelling suture (top) and schematic diagram (bottom) during MCAO modeling in mice. (D) Flow cytometric analysis of unlabeled or DiI‐labeled MSCs^HO‐1^ and in vivo fluorescence imaging in EP tubes (left 1, left 2). In vivo fluorescence images of MSCs or MSCs^HO‐1^ in major organs at 4, 8, and 24 h after injection of Dil‐labeled MSCs or MSCs^HO‐1^ through the tail vein of mice for 24 h (right 1, right 2, right 3). (E) Analysis of accumulation of DiI‐labeled MSCs^HO‐1^ in brain tissue by immunofluorescence, whole‐brain slice (left), partial enlargement of the injured area: DiI, DAPI, merge (right) from top to bottom. Scale bar, 100 μm.

Ischemic stroke was surgically established in mice by blocking the middle cerebral arteries. The experimental process is illustrated in Figure 4A. The success of the MCAO model was verified by TTC staining of brain slices (Figure 4B). MSC transplantation was performed via intravenous injection rather than stereotaxic intracranial injection because the procedure is safe, simple, and minimally invasive. MSCs were labeled with DiI cell membrane red fluorescent probes according to a previously reported method to track the in vivo distribution of intravenously injected stem cells. After incubation of HO‐1‐overexpressing MSCs with DiI for 30 min, more than 99% of the MSCs were labeled (Figure 4D). MSCs first appeared in the lungs, liver, and kidneys after intravenous injection.

### 3.4 MSCs^HO^ ^‐1^ increase neuronal cell survival and reduce microglia

**FIGURE 5:**
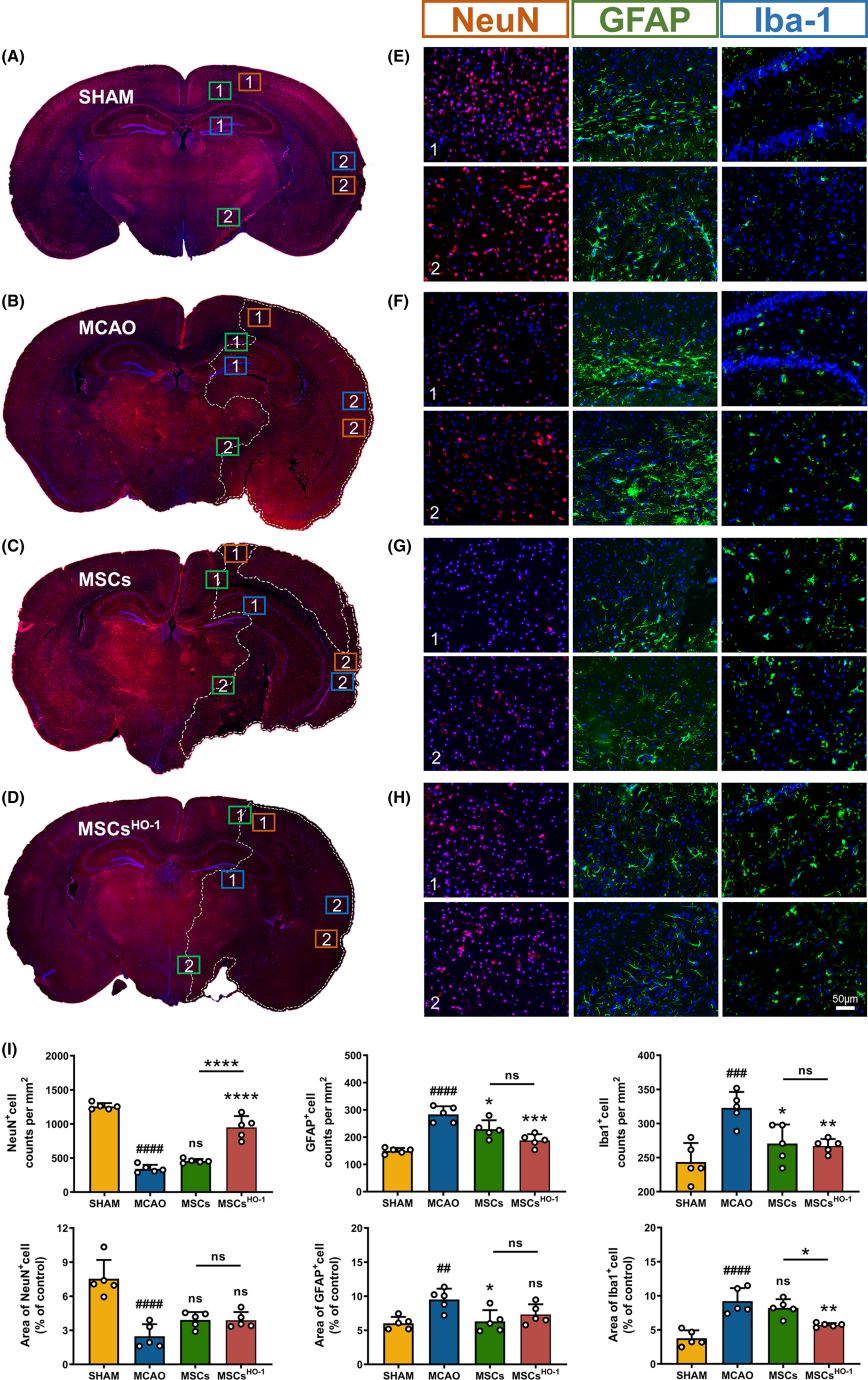
Immunofluorescence staining showed the differences in neurons and glial cells in different treatment groups. (A–H) Representative images of NeuN and DAPI staining at 10× magnification 4 days after middle cerebral artery occlusion (MCAO). Representative brain sections from the sham‐operated group as well as the four animal groups in the early injection of phosphate buffer saline, mesenchymal cells (MSCs), and MSCs^HO‐1^ are shown. The white line delineates the lesion area within the ischemic hemisphere. Scale bar 50 μm. (I) The number of cells per square millimeter and the relative fluorescence area of NeuN‐, GFAP‐, and Iba‐1‐positive cells in Figure 5 were counted (*n* = 5). Data are presented as the mean ± SD of five independent experiments. ^##^ *p* < 0.01, ^###^ *p* < 0.001, ^####^ *p* < 0.0001 MCAO vs. SHAM. *p < 0.05, **p < 0.01, ***p < 0.001, ****p < 0.0001, MSCs vs. MCAO, MSCs^HO‐1^ vs. MCAO, MSCs^HO‐1^ vs. MSCs. One‐way ANOVA followed by Bonferroni's tests.

The in vivo therapeutic effect of MSCs^HO‐1^ was assessed using the MCAO mouse model (Figure 4A). Immunofluorescence staining was performed to examine the spatiotemporal morphological changes in neurons, immune cells, and glial cells. Survival of mice is unstable during the acute inflammatory phase of ischemic injury. On the 3rd day after modeling, frozen sections of the mouse brain showed a significant reduction in the fluorescence signaling of NeuN‐labeled neurons in the infarcted area of MCAO mouse brains (Figure 5B,F,I, ^####^ *p* < 0.0001). In contrast, neuronal loss was reduced in the groups treated with intravenous infusion of hUC‐MSCs and hUC‐MSCs^HO‐1^ (Figure 5C,D,G,H), especially with the latter (Figure 5I, ****p < 0.0001). The fluorescence count of NeuN+ cells in the hUC‐MSCs^HO‐1^ group was similar to that in the sham group, but the average fluorescence area was smaller than that in the sham group. The number of surviving neurons is restored, whereas the smaller surviving neurons may be degenerate neurons. The closer the surviving microglia were to the ischemic core, the higher their activity. Astrocytes were significantly increased around the injury site, and the cells exhibited a hypertrophic morphology with longer protrusions. Quantitative analysis of the morphological differences between microglia, neurons, and astrocytes showed that in the ischemic hemisphere, the number of microglia/macrophages significantly increased after stroke induction. In contrast, the number of neurons significantly decreased (Figure 5I). Intravenous infusion of hUC‐MSCs and hUC‐MSCs^HO‐1^ decreased inflammation and increased neuronal survival.

### 3.5 Microglial M2 polarization after MSC^HO^ ^‐1^ treatment

**FIGURE 6:**
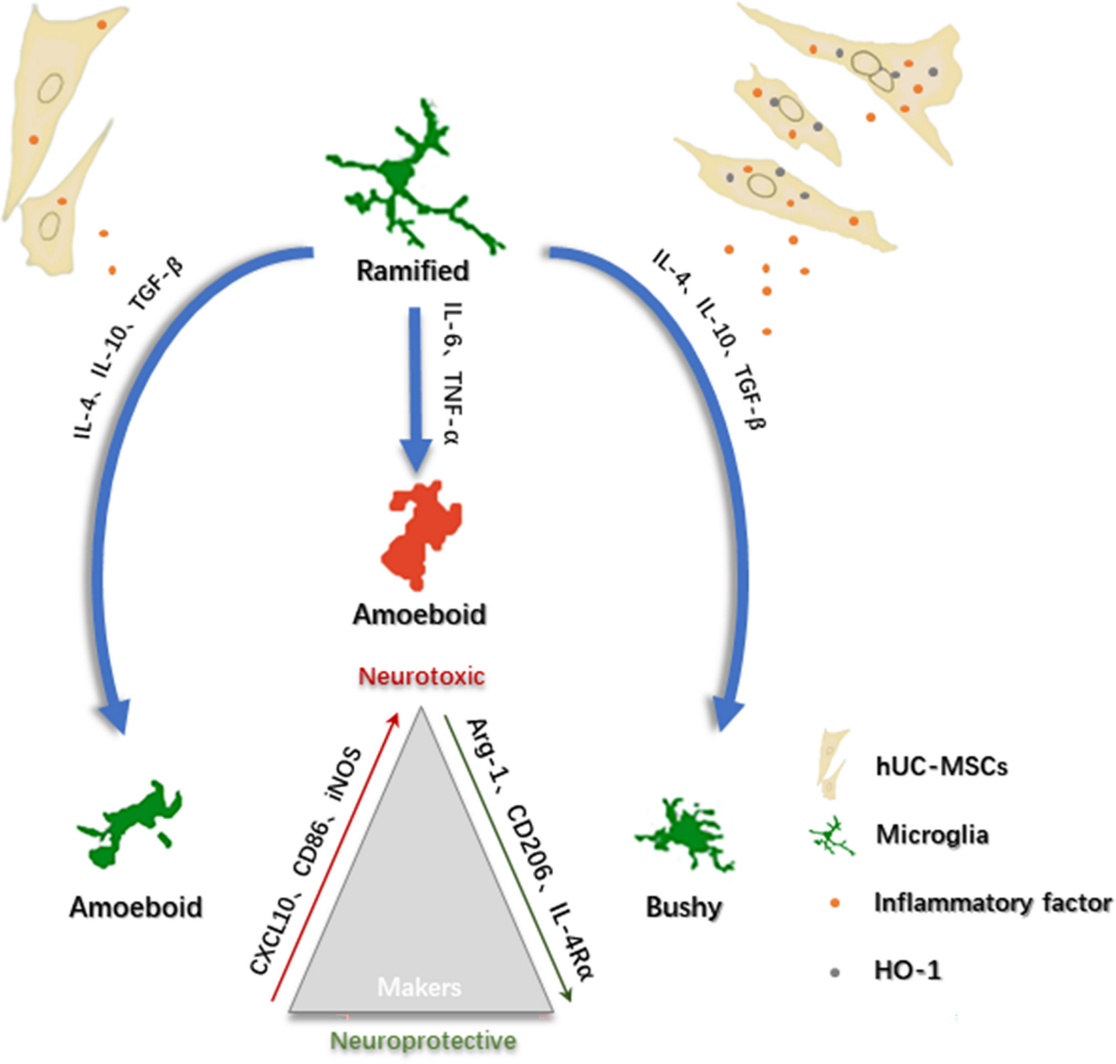
Protocol for the modulation of ischemic stroke injury by mesenchymal cell (MSC^HO‐1^) treatment. Immediately after ischemic stroke, microglia are hyperactivated and release potent inflammatory cytokines, such as IL‐1, IL‐6, and TNF‐α, leading to a severe inflammatory response that exacerbates brain damage. MSC^HO‐1^ treatment released more anti‐inflammatory cytokines (IL‐4, IL‐10, and TGF‐β) and decreased pro‐inflammatory cytokines (IL‐1β, IL‐6, TGF‐β) compared with MSC treatment. The expression and release of IL‐17 and TNF‐α can play a role in reducing excessive inflammatory activation injury by inhibiting the activation of microglial cells induced by ischemic stroke injury or increasing the transformation of microglial cells from the M1 type to the M2 type.

**FIGURE 7:**
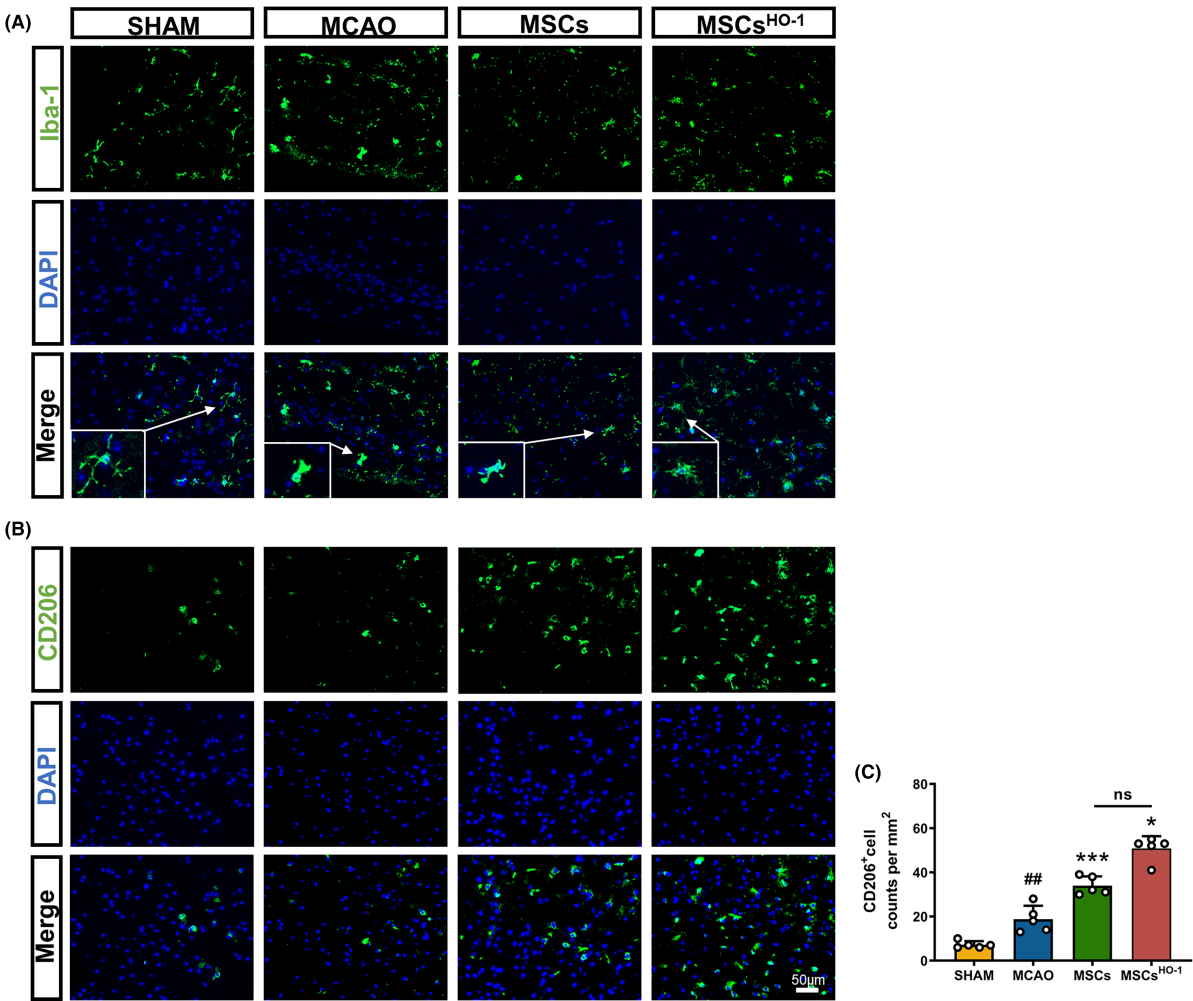
Representative images of Iba‐1 and CD206 staining 4 days after middle cerebral artery occlusion (MCAO). (A, B) Representative results of Iba‐1 and CD206 staining in the sham‐operated group and the four animal groups injected with phosphate buffer saline, mesenchymal stem cells (MSCs), and MSCs^HO‐1^ at an early stage. The typical microglial morphological features of the ischemic area are magnified in the white box. Scale bar 50 μm. (C) The number of CD206‐positive cells per square millimeter (*n* = 5) was counted. Data are presented as the mean ± SD of five independent experiments. ^##^ *p* < 0.01, MCAO vs. SHAM. *p < 0.05, ***p < 0.001, MSCs vs. MCAO, MSCs^HO‐1^ vs. MCAO, MSCs^HO‐1^ vs. MSCs. One‐way ANOVA followed by Bonferroni's tests.

In the Sham group, most branched cells were in a quiescent state. In contrast, the ischemic core area in the MCAO group consisted predominantly of activated round cells (Figure 6). In the ischemic area of hUC‐MSCs, the cells were deformed with a few round cells. The hUC‐MSC^HO‐1^ group consisted mostly of intermediate cells (Figure 7A).

**FIGURE 8:**
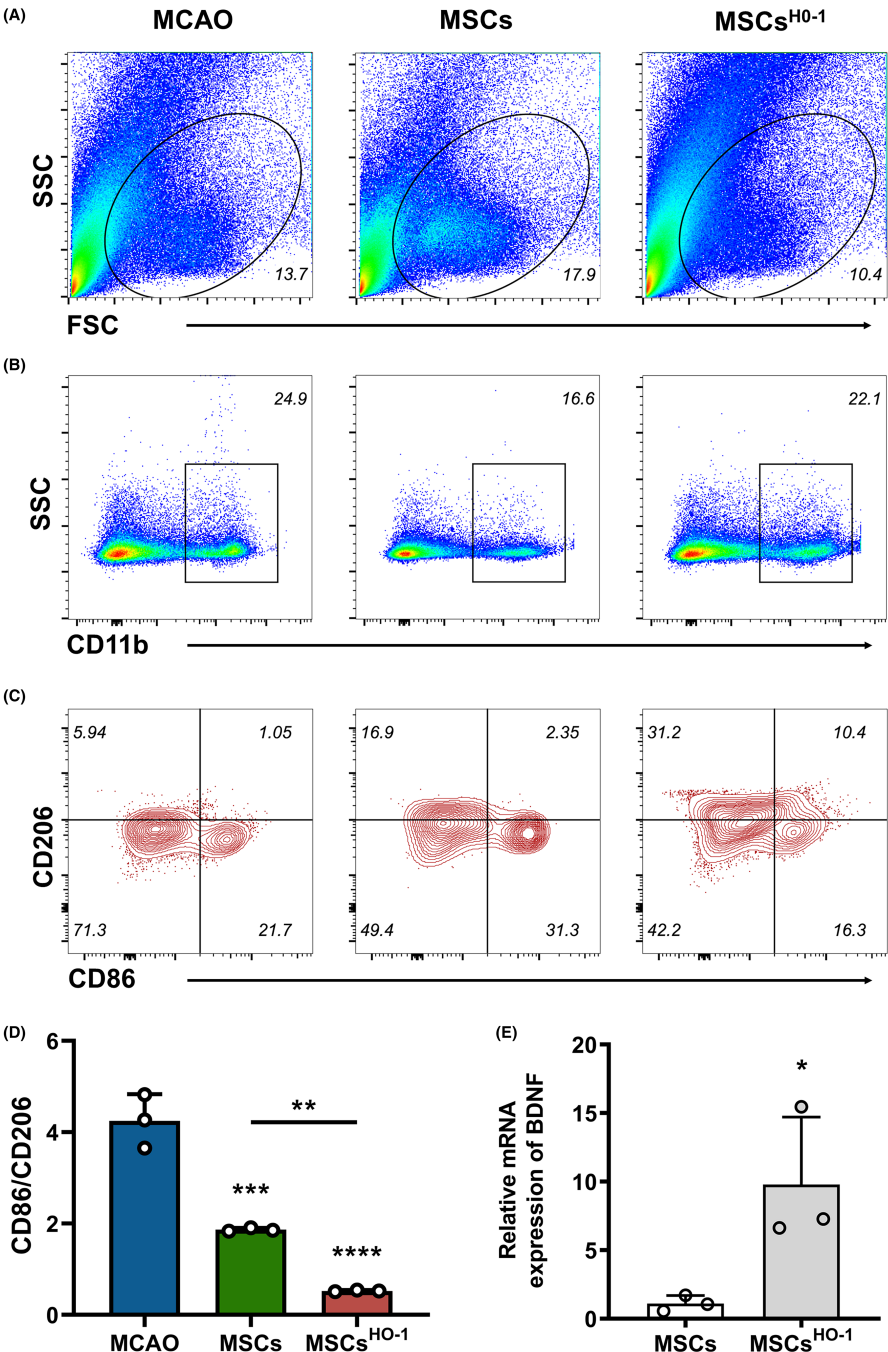
In the brains of middle cerebral artery occlusion (MCAO) mice following phosphate buffer saline/mesenchymal stem cells (MSC)/MSC^HO‐1^ treatment, activated microglia/macrophages were biased toward M2 polarization. (A) Graph showing the live cell gating strategy on roughly the same scale. (B) Graph showing the gating strategy for CD11b+ cells, denoted as microglia/macrophages in boxes. (C) Graph shows the population of CD86+ and CD206+ cells gated on CD11b+ cells. (D) Graph showing the calculated percentage of CD86+ CD206− and CD206+ CD86− cells of CD11b+ cells in each treatment group. Data are presented as the mean ± SD of three independent experiments. **p < 0.01, ***p < 0.001, ****p < 0.0001, MSCs vs. MCAO, MSCs^HO‐1^ vs. MCAO, MSCs^HO‐1^ vs. MSCs. One‐way ANOVA followed by Bonferroni's tests. (E) mRNA expression of brain‐derived neurotrophic factors (mRNA obtained from MSCs and MSCs^HO‐1^ cultured in vitro), *p < 0.05. Student's *t*‐test (*n* = 3).

The number of areas of ischemic injury in the MCAO, hUC‐MSC, and hUC‐MSC^HO‐1^ groups increased significantly (Figure 7B,C), indicating that inflammation in the hUC‐MSC group and the hUC‐MSC^HO‐1^ group was suppressed, especially in the hUC‐MSC^HO‐1^ group. We used this part of the experiment to assess whether altering the internal environment at the injury site after cell transplantation produced a more pronounced M2 phenotype. To further verify the inflammation‐inhibiting properties of hUC‐MSCs^HO‐1^, flow cytometry was performed on single‐cell suspensions of whole ischemia‐injured brain tissue, showing that compared with the MCAO group, the CD86/CD206 ratio of the hUC‐MSC group was significantly lower. The CD86/CD206 ratio in the hUC‐MSC^HO‐1^ group was significantly lower than in the hUC‐MSC group. CD86 is considered a classic M1 marker, and CD206 is considered an M2 marker. In the hUC‐MSC group, the CD86/CD206 ratio was greater than 1, and M1 microglia were predominant (Figure 8A–C). The CD86/CD206 ratio in the hUC‐MSC^HO‐1^ group was <1 (0.52), and M2 microglia were dominant (Figure 8D).

### 3.6 Evaluation of the therapeutic effect of MSCs^HO^ ^‐1^ in vivo

**FIGURE 9:**
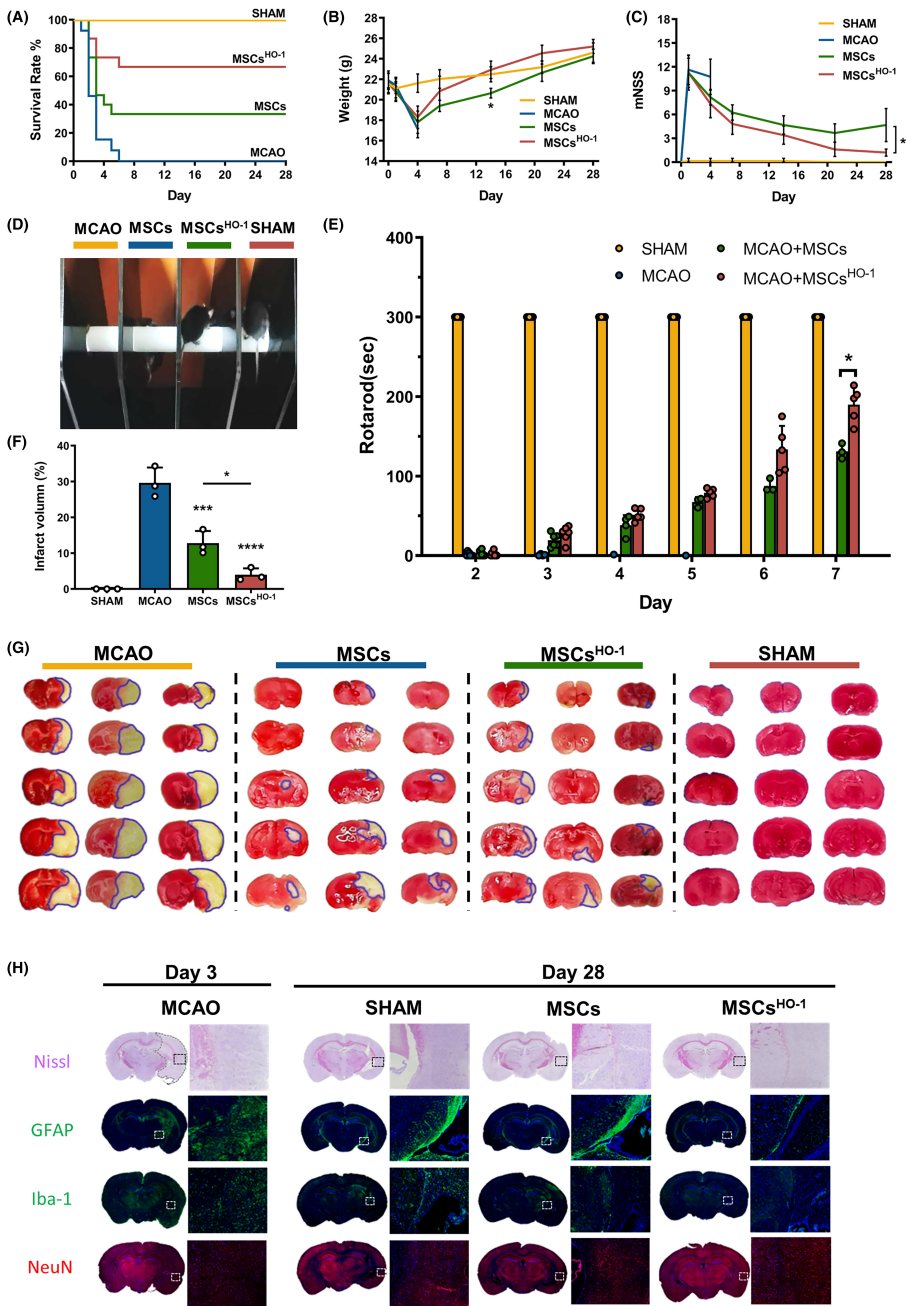
Therapeutic effect of heme oxygenase‐1 (HO‐1)‐transduced mesenchymal stem cells on ischemic stroke. (A) Survival of middle cerebral artery occlusion (MCAO) mice receiving different treatments (*n* = 15), mesenchymal stem cells (MSCs) (3 × 10^5^), HO‐1‐transduced MSCs (MSCs^HO‐1^, 3 × 10^5^) or phosphate buffer saline. Body weight measurements and behavioral tests were performed on Days 1, 4, 7, 14, 21, and 28 after MCAO induction. (B) Body weight (*n* = 8), *p < 0.05, MSCs^HO‐1^ vs. MSCs (Day 14), two‐way ANOVA followed by Bonferroni's test, and (C) mean mNSS scores in MCAO mice during treatment. A higher score indicates a more serious injury. *p < 0.05, MSCs^HO‐1^ vs. MSCs (the whole group), two‐way ANOVA followed by Bonferroni's test. (D) Representative images of the rotarod test on Day 4 of mice in different treatment groups. (E) Rotarod test statistic (*n* = 8). *p < 0.05, MSCs^HO‐1^ vs. MSCs (Day 7), two‐way ANOVA followed by Bonferroni's test. (F) Statistical plot of relative values of ischemic volume (*n* = 3), *p < 0.05, ***p < 0.001, ****p < 0.0001, MSCs vs. MCAO, MSCs^HO‐1^ vs. MCAO, MSCs^HO‐1^ vs. MSCs. One‐way ANOVA followed by Bonferroni's tests, (G) ischemic volume determined by 2,3,5‐triphenyltetrazolium chloride staining, blue curve in TTC staining. The boxed area represents the infarct area. (H) Representative images of pathological examination of MCAO mouse brain sections, including Nissl staining, glial fibrillary acidic protein (GFAP) staining (green, representing astrocytes), ionic calcium‐binding adaptor 1 (Iba‐1) staining (green, for microglia), and NeuN staining (red, for neurons).

The MCAO mice could not survive for more than 5 days without treatment. Transplantation of hUC‐MSCs improved the survival rate of the MCAO mice by approximately 33%. Remarkably, hUC‐MSC^HO‐1^ treatment significantly improved the survival rate of mice by approximately 64% (Figure 9A). In addition, the body weight of MCAO mice treated with hUC‐MSCs or hUC‐MSCs^HO‐1^ declined sharply in the first 4 days and then recovered gradually; however, MCAO mice treated with hUC‐MSCs^HO‐1^ recovered faster than the hUC‐MSC‐treated group, exhibiting normal levels on Day 14 (Figure 9B, *p < 0.05). These results demonstrate that HO‐1‐overexpressing umbilical cord stem cells are more effective than hUC‐MSCs in reducing mouse mortality after MCAO development.

The mNSS assessed functional recovery in treated mice, which reflects motor function, sensory acuity, balance, and reflexes in MCAO mice. The scores of the hUC‐MSC^HO‐1^ mice after ischemic–reperfusion were significantly lower than those of the MSC‐treated group (Figure 9C, *p < 0.05, mixed‐effect analysis of variance). MCAO mice treated with hUC‐MSCs^HO‐1^ stayed longer on the rotarod than mice treated with hUC‐MSCs [Figure 9D,E; an additional movie file shows this in more detail in File S7)]. Moreover, the rotarod data showed that hUC‐MSCs^HO‐1^ was more effective in restoring brain function than hUC‐MSCs within a week (Figure 9E, *p < 0.05).

The ischemic brain area after hUC‐MSC^HO‐1^ or hUC‐MSC treatment was determined by 2,3,5‐triphenyltetrazolium chloride (TTC) staining (white area in Figure 9G). TTC staining (Figure 9G) and Nissl staining (Figure 9H) of the ischemic brain showed that hUC‐MSCs^HO‐1^ and hUC‐MSCs had obvious protective effects on brain cells compared with large‐area ischemia in the MCAO group (Figure 9F, ****p < 0.0001, ***p < 0.001). Notably, mice treated with hUC‐MSCs^HO‐1^ had fewer astrocytes but more neurons than the hUC‐MSC‐treated group. This observation indicates a better recovery of neuronal function with hUC‐MSCs^HO‐1^. Additionally, fewer microglia were observed, as previously described, demonstrating a more pronounced inhibition of inflammatory damage (Figure 9H).

### 3.7 Levels of cytokines and BDNF after MSC^HO^ ^‐1^ treatment in vivo

**FIGURE 10:**
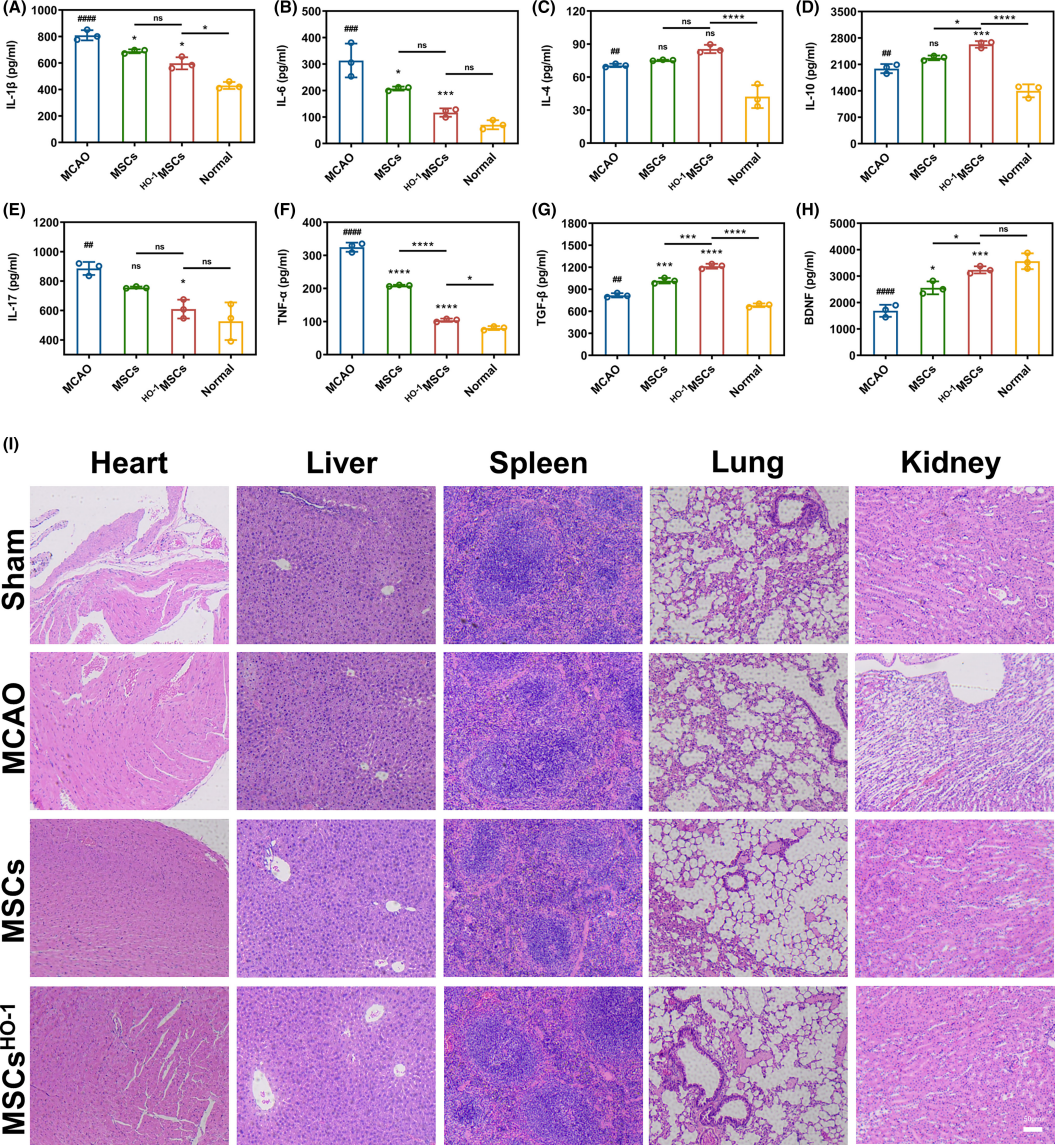
ELISA for the factors. (A) IL‐1β, (B) IL‐6, (C) IL‐4, (D) IL‐10, (E) IL‐17, (F) TNF‐α, (G) TGF‐β, and (H) BDNF 3 days after middle cerebral artery occlusion (MCAO) operation. Data are presented as the mean ± SD of three independent experiments. ^##^ *p* < 0.01, ^###^ *p* < 0.001, ^####^ *p* < 0.0001, MCAO vs. Normal. *p < 0.05, ***p < 0.001, ****p < 0.0001, mesenchymal stem cells (MSCs) vs. MCAO, MSCs^HO‐1^ vs. MCAO, MSCs^HO‐1^ vs. Normal, MSCs^HO‐1^ vs. MSCs. One‐way ANOVA followed by Bonferroni's tests. (I) Safety evaluation of MSCs^HO‐1^ in vivo. Paraffin‐embedded tissue sections were stained with hematoxylin and eosin for light microscopy assessment (*n* = 4). Scale bar, 50 μm.

Finally, the ELISA results on the 3rd day after tissue homogenization of the mice in each group showed that hUC‐MSCs^HO‐1^ reduced the expression of pro‐inflammatory factors (IL‐1β, IL‐6, IL‐17, and TNF‐α) and increased the expression of anti‐inflammatory factors (IL‐4, IL‐10, and TGF‐β) and BDNF. Primitive MSCs secreted BDNF slowly, whereas lentivirus‐transduced MSCs (hUC‐MSCs^HO‐1^) secreted more BDNF (Figure 8E). The expression levels of inflammatory factors and BDNF in the brains of MCAO mice were compared after MSC treatment. Twenty‐four hours after modeling, lentivirus‐transduced hUC‐MSCs or hUC‐MSCs were injected intravenously into MCAO mice. The brains of the MCAO mice exhibited higher levels of IL‐1β (Figure 10A, *p < 0.05), IL‐6 (Figure 10B, *p < 0.05), and TNF‐α (Figure 10F, ****p < 0.0001) and lower levels of TGF‐β (Figure 10G, ***p < 0.001) than hUC‐MSC‐treated mice. The brains of hUC‐MSC‐treated mice exhibited higher levels of TNF‐α (Figure 10F, ****p < 0.0001) than hUC‐MSC^HO‐1^‐treated mice, while IL‐10 (Figure 10D, *p < 0.05) and TGF‐β (Figure 10G, ***p < 0.001) were lower. In contrast, hUC‐MSC^HO‐1^‐treated mice had higher levels of IL‐1β (Figure 10A, *p < 0.05), IL‐4 (Figure 10C, ****p < 0.0001), IL‐10 (Figure 10D, ****p < 0.0001), TNF‐α (Figure 10F, *p < 0.05), and TGF‐β (Figure 10G, ****p < 0.0001) in the brain than the normal group. The BDNF levels in the brains of MCAO mice were much lower (56%) than those in normal mice on Day 2 after modeling. Compared to control MCAO mice, hUC‐MSC treatment increased BDNF levels in the brain, although they remained lower than those with hUC‐MSCs^HO‐1^, which yielded increased brain BDNF levels in MCAO mice, close to the levels in normal mice (Figure 10H).

### 3.8 In vivo safety assessment of MSCs

Finally, we evaluated the in vivo safety of the hUC‐MSCs^HO‐1^. Histological sections of the paraffin‐embedded heart, liver, spleen, lungs, and kidneys were stained with hematoxylin and eosin (HE) for light microscopy evaluation (Figure 10I). The HE imaging results were all normal, and there was no tumorigenesis (uncontrolled cell proliferation) of the engineered transplanted cells, as mentioned in the literature.^25^

## 4 DISCUSSION

It has been reported that the expression of HO‐1 or the application of its important product CO mediates a strong anti‐inflammatory effect in monocytes, macrophages (MF), or both which may inhibit the ability of these cells to induce tissue damage and modulate their role in initiating immune responses.^26^ Microglia are macrophages in the brain tissue increasingly recognized as key players in CNS development and homeostasis.^27^ Microglia are essential for maintaining normal brain function, and their morphology is closely related to their function. Under normal conditions, resting microglia have small cell bodies and highly branched cytoplasm. When brain homeostasis is disrupted, such as during CNS injury or infection, microglia are activated and become chemotactic toward the injury site. Simultaneously, the cell body enlarged, the processes retracted, and the cells were round or rod‐shaped. Subsequently, the activated microglia were further activated and adjusted, the processes disappeared, the morphology became amoeboid, and they exhibited a phagocytic clearance function. Morphological changes in microglia reflect their activation state, which is closely related to the severity of the damaged site in the brain.^28^ Microglia are classified into branched, intermediate, deformed, and round phenotypes. The severity of ischemia is related to the morphology of these microglia.^26^

In the inflammatory state, microglia show not only morphological differences as described above. Cell phenotypes vary considerably at the microscopic level. Two different polarization phenotypes were observed in infiltrating macrophages and brain microglia (M1 and M2), reflecting the inflammatory response's duality. Both cell types exhibit a spatiotemporal transition from an early beneficial M2 phenotype (alternate activation) to a long‐term deleterious M1 phenotype (classical activation).^29^, ^30^ The mannose receptor CD206 is an important pattern recognition and endocytic receptor in the innate immune system that can recognize and bind a wide range of endogenous and exogenous ligands. It is important in maintaining homeostasis, identifying pathogens, inducing cytokines, antigen presentation, and other processes (Figure 7).^31^, ^32^ In this study, owing to the inhibition of microglial polarization, mice treated with MSC^HO‐1^ overexpression experienced a more significant inhibition of inflammatory damage and better nerve function restoration.

We also focused on the broad application prospects of MSCs^33^ and engineered hUC‐MSCs overexpressing HO‐1. Transduction of HO‐1 leads to local anti‐inflammatory effects in hUC‐MSCs and provides nutrition to nerve cells to improve nerve function.^34^, ^35^ Many cells accumulate in the damaged tissue after intravenous injection, and tissue damage repair and regeneration can be promoted through cell replacement, microenvironment communication, or enhancement of the regeneration ability. In addition, stimulated by inflammatory signals, MSCs produce various growth factors that promote the repair of damaged tissues by promoting angiogenesis, extracellular matrix (ECM) remodeling, and differentiation of tissue precursor cells.

Our study provides compelling evidence that MSCs overexpressing HO‐1 can effectively regulate microglial polarization in the tissue microenvironment during inflammation. The type and intensity of inflammation determine the immunomodulatory capacity of MSCs. Several preclinical and clinical studies have substantiated the favorable regenerative role of MSCs in pathological conditions characterized by inflammation, including graft‐versus‐host disease (GvHD),^36^, ^37^ multiple sclerosis,^38^ and diabetic nephropathy.^39^ Our study revealed that the therapeutic effect of MSCs overexpressing HO‐1 was significant in cerebral ischemia–reperfusion injury. Thus, the polarization of macrophages/microglia from a pro‐inflammatory to an anti‐inflammatory phenotype inhibits inflammation. In addition to microglia, which are inherent to the CNS, it is highly conceivable that peripheral immune cells also play an important role in the injury process. Studies on MSC transplantation into animals or humans with inflammatory diseases have shown that MSCs have a strong inhibitory effect on the activation and proliferation of T cells and T cell‐mediated inflammatory responses. There is a growing consensus that the immunosuppressive capacity of MSCs is not structural but rather permitted by inflammatory cytokines in the microenvironment.^40^ Engineered MSCs exhibited good performance in regulating inflammatory cytokines in the microenvironment; however, their nonstructural anti‐inflammatory ability in the CNS remains to be explored.^18^ This investigation was also a missing part of the experimental procedure.

There is a lack of effective means for addressing injury and post‐injury recovery after stroke.^3^ Cell therapy is the solution we have been looking for, and stem cells are the best option. Although studies have shown that MSC‐derived exocrine vesicles yield therapeutic effects, they are not easy to obtain, and their targeting abilities remain unclear.^41^ Current evidence shows that stem cells exert their regenerative therapeutic effects through their differentiation capacity and secretion of neurotrophic factors, of which BDNF plays an important role in ischemic brain tissue repair.^42^ Therefore, MSC therapy is a reliable modality for CNS regeneration. We substantiated that intravenously infused MSCs were initially abundant in the lungs, liver, and kidneys, presumably due to a more abundant blood supply to these organs.^18^ Eventually, they were abundantly enriched in the brain ischemic injury areas within 24 h. These findings indicate that HO‐1 lentivirus‐transduced hUC‐MSCs can migrate and survive in ischemic areas and efficiently secrete BDNF. Our attempt to investigate whether infused MSCs have the potential to differentiate into neural cells was ultimately shelved because of insufficient evidence, given that even low‐immunogenicity MSCs are subject to mouse immune elimination when they enter the body.^43^ Interestingly, we could no longer track RFP‐expressing MSCs after 2–4 weeks; however, in the in vitro experiments, we mimicked the process of inducing neuroblasts and confirmed their differentiation‐inducing abilities (File S8). Future studies should focus on escaping host immunity and achieving successful host differentiation in vivo.

Here, we explored the immunomodulatory, neuroprotective, and tissue repair properties of MSCs, which account for their beneficial effects during post‐stroke recovery. To alleviate inflammation and restore neurological function, we introduced HO‐1, a molecule with potent antioxidant, anti‐injury, immunomodulatory, and neurotrophic effects. Our study showed that MSC overexpression increased the expression of neurotrophic and anti‐inflammatory factors and decreased the expression of pro‐inflammatory factors in vitro and in vivo. This effect was reflected in the MCAO mouse brain by reversing the polarization of M1 microglia to M2 microglia. Overexpression of HO‐1 also decreases the number and activation of astrocytes and reduces the production of glial scars, hindering synaptic communication in nerve cells. Simultaneously, increased BDNF levels nourished neurons, improved neuronal damage, and enhanced neurological recovery. Ultimately, an excellent therapeutic effect was achieved, and the survival rate of MCAO mice was improved.

## 5 CONCLUSION

HO‐1 lentivirus was used as a vector for efficient gene transduction in hUC‐MSCs. As a result, HO‐1‐transduced MSCs secreted more BDNF and anti‐inflammatory factors and fewer pro‐inflammatory factors in the ischemic brain region in vitro and in vivo, thereby significantly reducing the mortality of MCAO mice and reducing the secondary injury caused by inflammation to improve nerve reconstruction and motor function. These results provide a theoretical basis for improving clinical interventions and rehabilitation treatments for ischemic stroke.

## AUTHOR CONTRIBUTIONS

YY contributed to the conception and design, data analysis, and manuscript writing; QQL contributed to data collection and analysis; SD, QS, LP, YJL, YH, SQZ, and QJJ performed the research; JC and DKN contributed to design, financial support, and final approval of the manuscript. All authors read and approved the final manuscript.

## FUNDING INFORMATION

This work was supported by Nantong Science and Technology Plan Project (MS22020017, JC2020060); Yancheng Medical Science and Technology Development Plan Project (YK2015006); Nantong “Fourteenth Five Year Plan” Science and Education Strong Health Engineering Medical Innovation Team.

## CONFLICT OF INTEREST STATEMENT

The authors declare that they have no competing interests.

## Supporting information


File S1.
File S2.
File S3.
File S4.
File S5.
File S6.
File S7.
File S8.

Data S1.

## Data Availability

The data that support the findings of this study are available from the corresponding authors upon reasonable request.
