# Supplementary material for: Human umbilical cord derived mesenchymal stem cells overexpressing HO‐1 attenuate neural injury and enhance functional recovery by inhibiting inflammation in stroke mice
Source: CNS Neurosci Ther. 2023 Aug 17;30(2):e14412. doi: 10.1111/cns.14412 (PMC10848045; doi:10.1111/cns.14412)
Supplement: Supplementary file 1 — File S1. File S2. File S3. File S4. File S5. File S6. File S7. File S8. [file CNS-30-e14412-s002.zip › Supplementary Material/Additional file 1.rtf]

Antibody	Vendor	Application and dilution	CloneNo.	Cat No.	
Rabbit anti-HO-1 antibody	Abcam	IF: 1:500, 
WB: 1:20000	EPR1390Y	ab68477	
Rabbit anti-DCX antibody	Proteintech	IF: 1:500	Polyclonal	13925-1-AP	
Mouse anti-â-Actin-Peroxidase antibody	Sigma	WB: 1:50000	AC-15	A3854	
Rabbit anti-NeuN antibody	Novus	IF: 1:500	2F9F0	NBP3-15656	
Mouse anti-GFAP antibody	Proteintech	IF: 1:1000	4B2E10	60190-1-Ig	
Rabbit anti-Iba-1 antibody	Wako	IF: 1:500	Polyclonal	019-19741	
Rabbit anti-CD206 antibody 	Proteintech	IF: 1:500	Polyclonal	18704-1-AP	
Rabbit anti-CD86 antibody 	Proteintech	IF: 1:100	Polyclonal	13395-1-AP	
CD11b Monoclonal Antibody, PE	eBioscience	FACS: 1:320	M1/70	12-0112-82	
APC/Cy7 anti-mouse CD86 Antibody	BioLegend	FACS: 1:200	GL-1	105029	
CD206 Monoclonal Antibody, APC	eBioscience	FACS: 1:160	MR6F3	17-2061-82	
FITC anti-human CD45 Antibody	BioLegend	FACS: 1:200	HI30	304006	
PE anti-human CD29 Antibody	BioLegend	FACS: 1:200	TS2/16	303004	
PE anti-human CD73 Antibody	BioLegend	FACS: 1:200	AD2	344004	
PE anti-human CD105 Antibody	BioLegend	FACS: 1:200	43A3	323206	
APC anti-human CD90 Antibody	BioLegend	FACS: 1:200	5E10	328114	
PerCP/Cy5.5 anti-human CD34 Antibody	BioLegend	FACS: 1:200	581	343522	
Anti-rabbit secondary antibodies	Beyotime	WB: 1:2000	None	A0208	
Alexa Fluor®488 Goat Anti-Mouse IgG	Invitrogen	IF: 1:1000	Polyclonal	A-11001	
Alexa Fluor®594 Goat Anti-Rabbit IgG	Invitrogen	IF: 1:1000	Polyclonal	A-11012	
Supplementary Table 1. Antibodies employed in this study.
