# Supplementary material for: Human umbilical cord derived mesenchymal stem cells overexpressing HO‐1 attenuate neural injury and enhance functional recovery by inhibiting inflammation in stroke mice
Source: CNS Neurosci Ther. 2023 Aug 17;30(2):e14412. doi: 10.1111/cns.14412 (PMC10848045; doi:10.1111/cns.14412)
Supplement: Supplementary file 1 — File S1. File S2. File S3. File S4. File S5. File S6. File S7. File S8. [file CNS-30-e14412-s002.zip › Supplementary Material/Additional file 3.docx]

**Supplementary Table 3.** Elisa kit in this study.

| **Name** | **Cat No.** | **Lot No.** | **Vendor** |
| --- | --- | --- | --- |
| TNF alpha Mouse Uncoated ELISA Kit | 88-7324-88 | 273586004 | Invitrogen |
| IL-6 Mouse Uncoated ELISA Kit | 88-7064-88 | 268362001 | Invitrogen |
| Mouse IL-1 beta Uncoated ELISA | 88-7013-88 | 267273001 | Invitrogen |
| IL-10 Mouse Uncoated ELISA Kit | 88-7105-88 | 263533002 | Invitrogen |
| Human/Mouse/Rat TGF-β1 ELISA Kit | EK981 | A98120515 | MultiSciences |
| Mouse IL-17A ELISA Kit | EK217/2 | A21711111 | MultiSciences |
| Mouse IL-4 ELISA Kit | EK204/2 | A20420124 | MultiSciences |
| Human BDNF ELISA Kit | EK1127 | A112710714 | MultiSciences |
