# Supplementary figures and images for: Human umbilical cord derived mesenchymal stem cells overexpressing HO‐1 attenuate neural injury and enhance functional recovery by inhibiting inflammation in stroke mice
Source: CNS Neurosci Ther. 2023 Aug 17;30(2):e14412. doi: 10.1111/cns.14412 (PMC10848045; doi:10.1111/cns.14412)

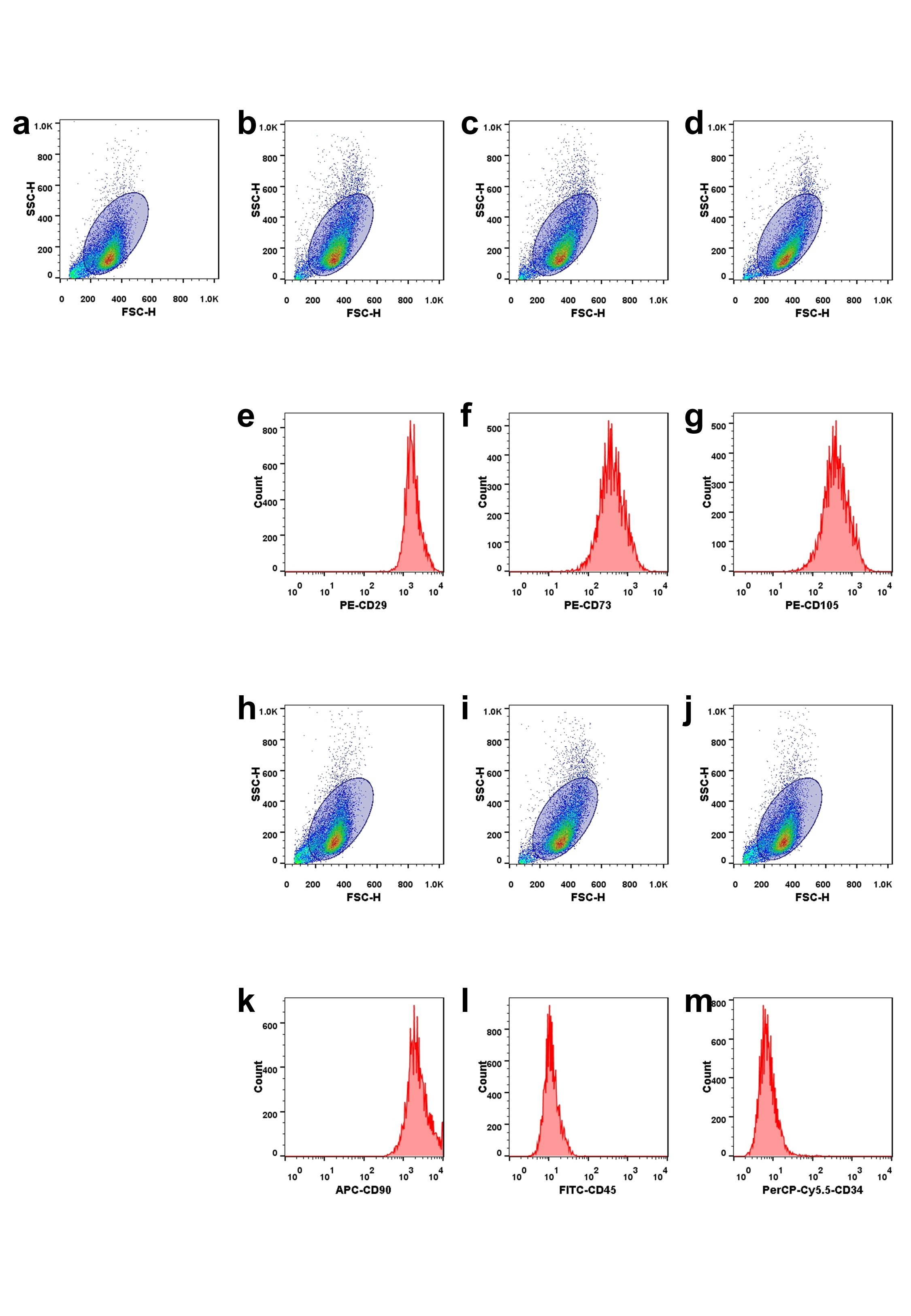

Supplement: Supplementary file 1 — File S1. File S2. File S3. File S4. File S5. File S6. File S7. File S8. [file CNS-30-e14412-s002.zip › Supplementary Material/Additional file 6.tif]

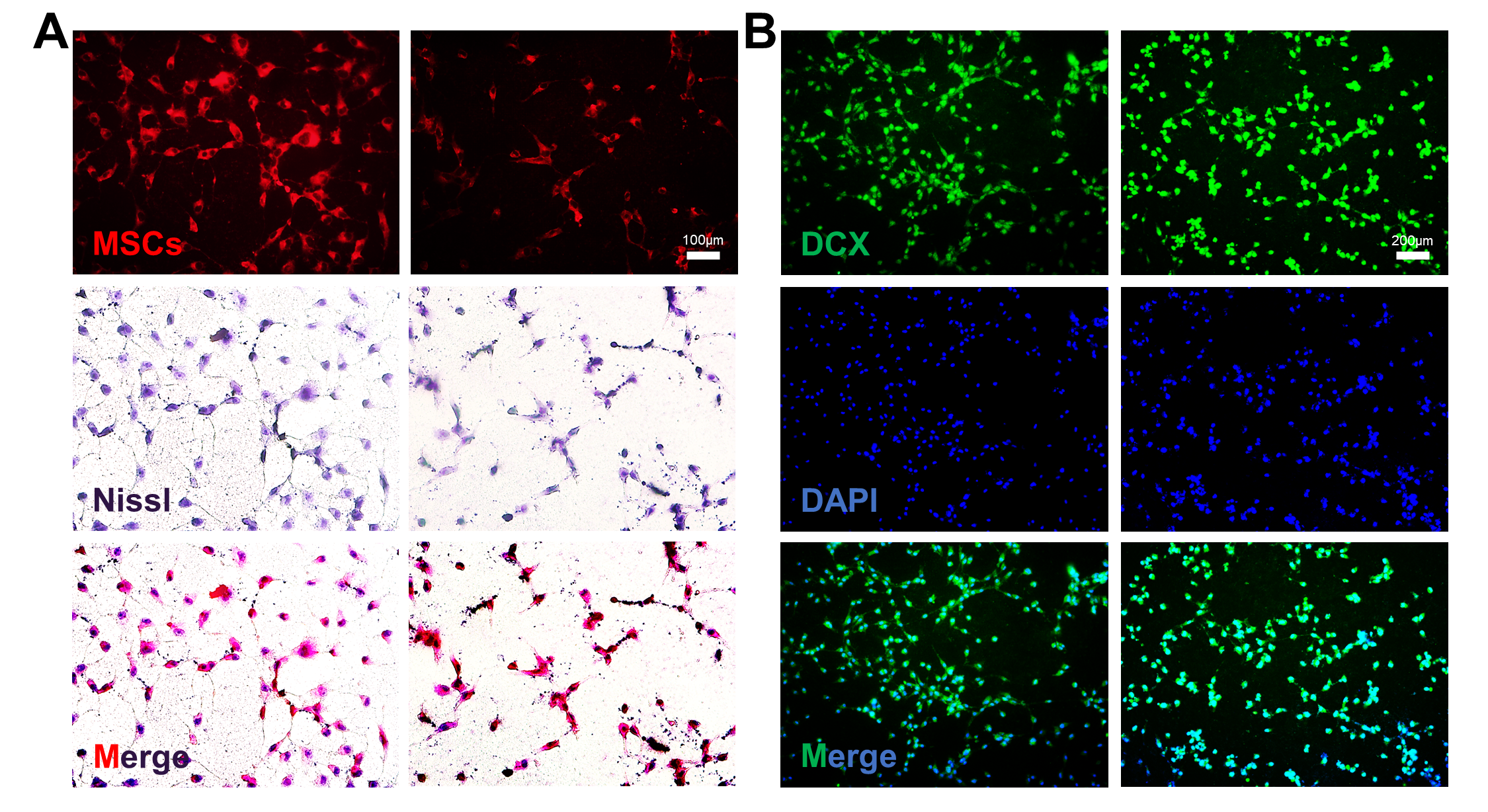

Supplement: Supplementary file 1 — File S1. File S2. File S3. File S4. File S5. File S6. File S7. File S8. [file CNS-30-e14412-s002.zip › Supplementary Material/Additional file 8.TIF]

Figure 2-A

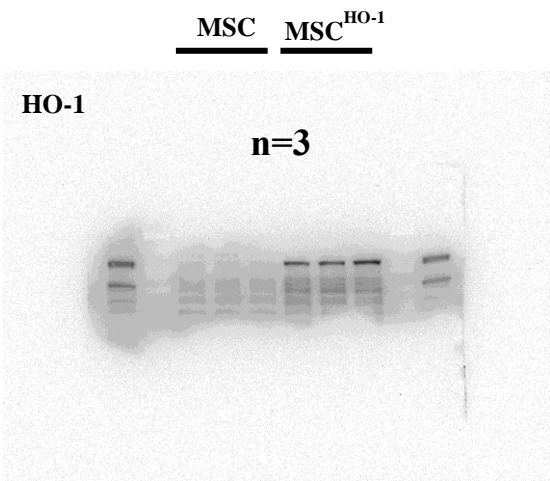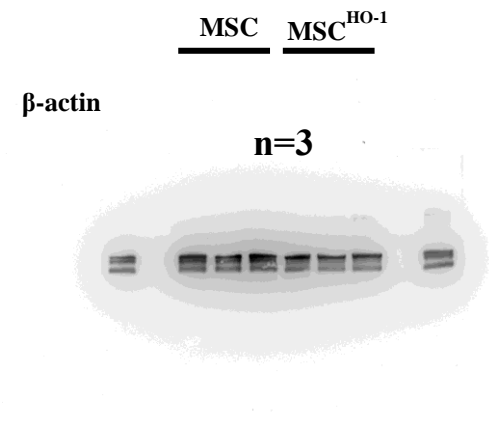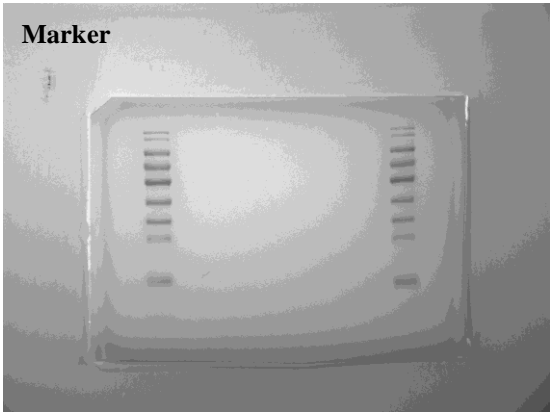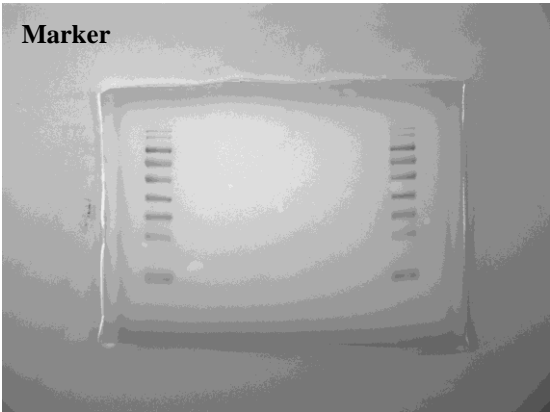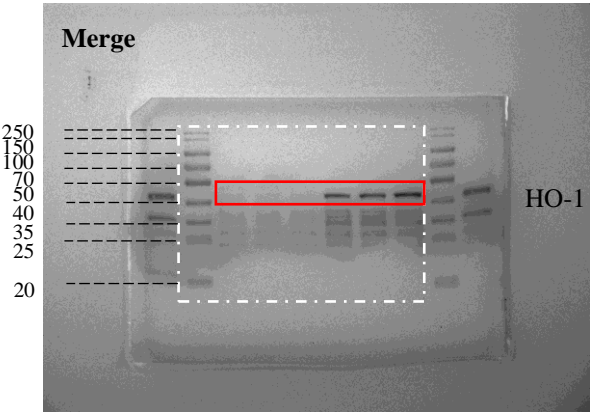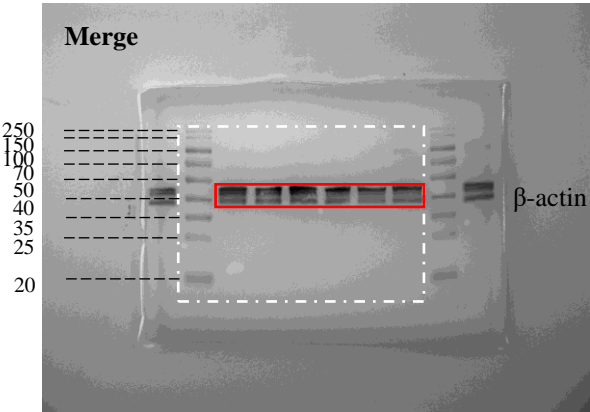

Supplement: Supplementary file 2 — Data S1. [file CNS-30-e14412-s001.zip › Full unedited blot for Figure 2A.pdf]
