# Supplementary material for: Human umbilical cord derived mesenchymal stem cells overexpressing HO‐1 attenuate neural injury and enhance functional recovery by inhibiting inflammation in stroke mice
Source: CNS Neurosci Ther. 2023 Aug 17;30(2):e14412. doi: 10.1111/cns.14412 (PMC10848045; doi:10.1111/cns.14412)
Supplement: Supplementary file 2 — Data S1. [file CNS-30-e14412-s001.zip › Original image.docx]

Original image

CD206 (Fig. 7C)


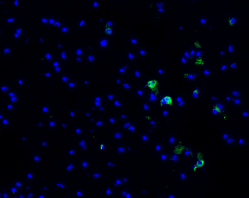

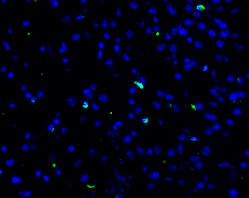

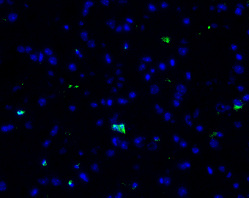

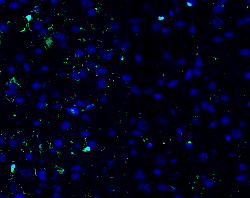

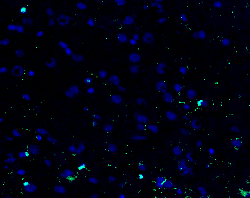

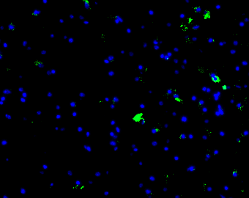

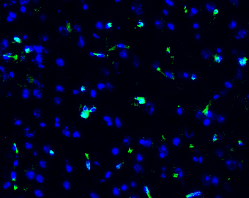

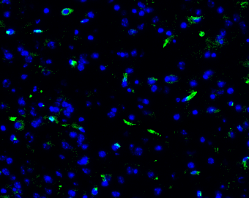

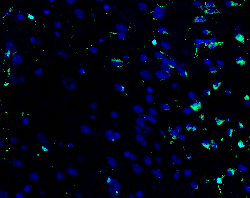

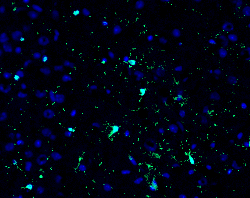

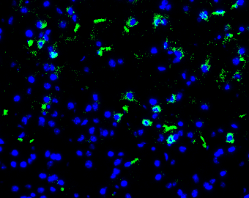

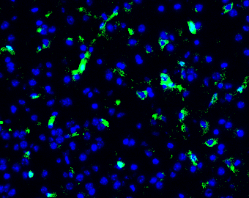

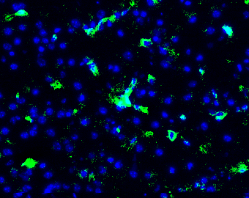

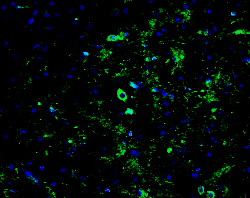

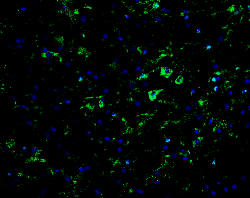

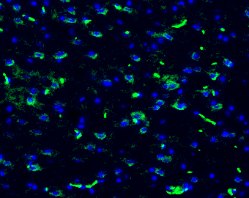

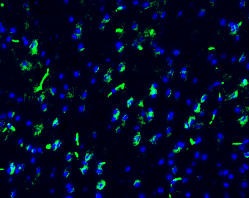

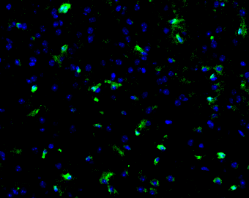

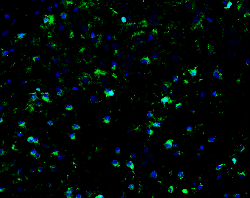

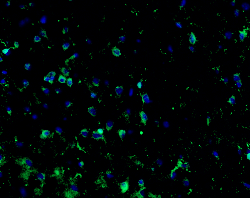
Total of four rows, from top to bottom, represent the Sham, MCAO, MSC, MSCHO-1 groups, and each group of 5 represents images

IBA-1 (Fig. 5I)


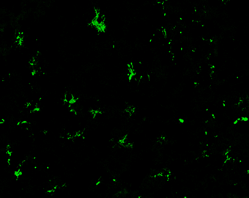

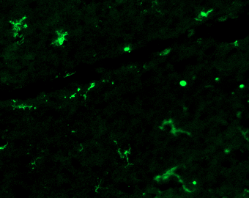

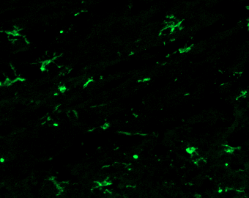

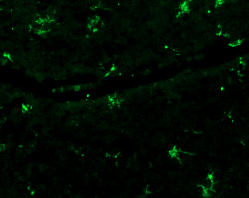

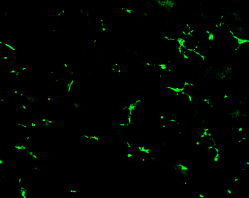

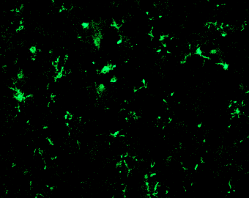

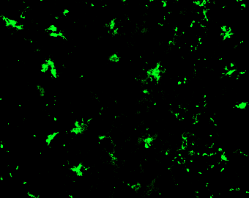

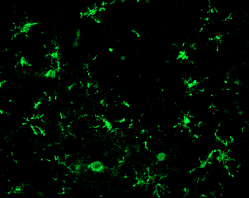

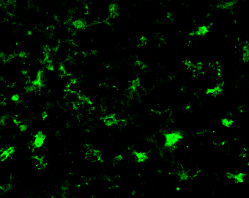

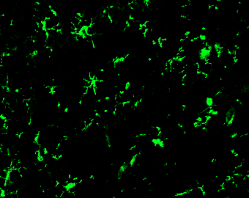

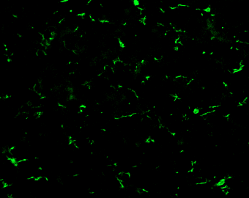

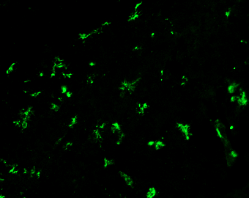

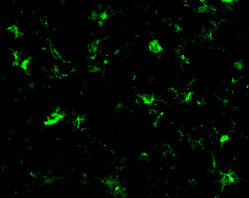

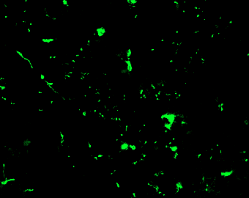

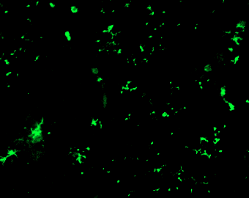

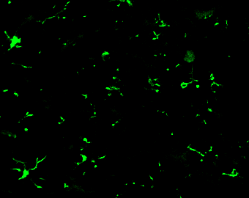

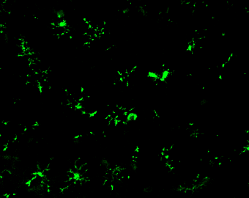

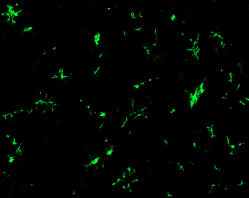

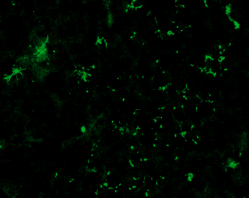

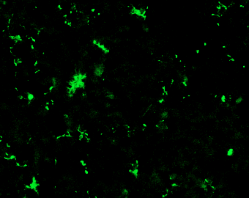
Total of four rows, from top to bottom, represent the Sham, MCAO, MSC, MSCHO-1 groups, and each group of 5 represents images

GFAP (Fig. 5I)


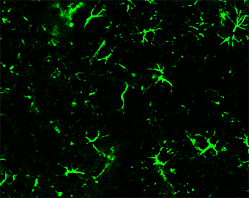

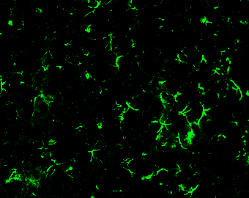

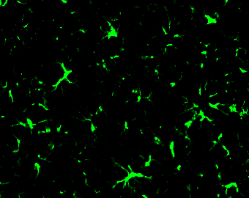

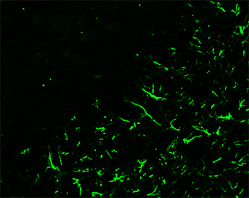

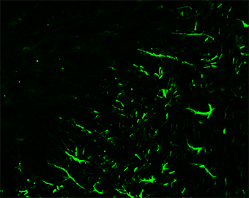

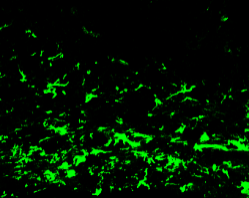

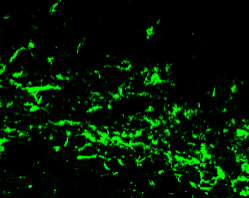

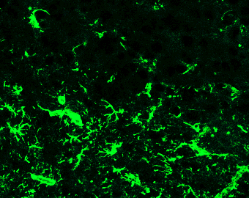

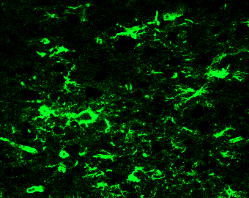

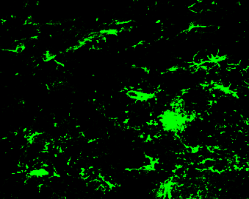

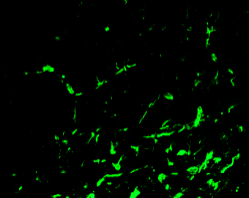

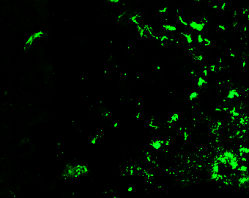

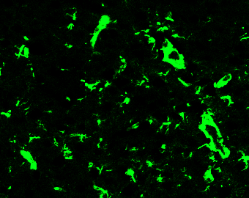

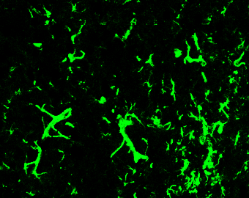

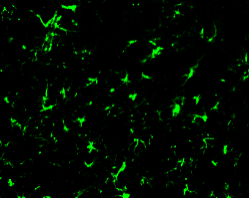

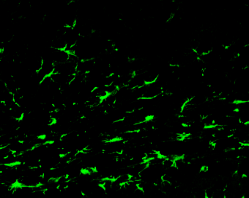

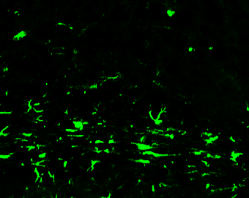

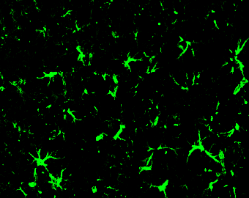

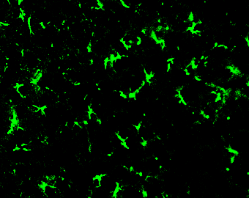

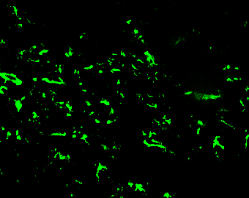
Total of four rows, from top to bottom, represent the Sham, MCAO, MSC, MSCHO-1 groups, and each group of 5 represents images

NeuN (Fig. 5I)


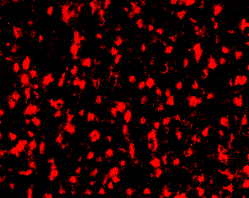

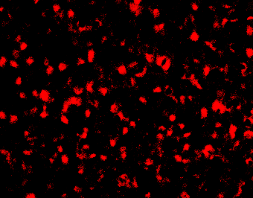

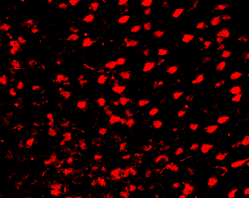

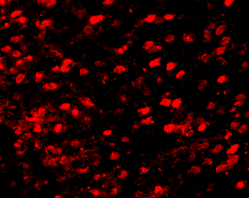

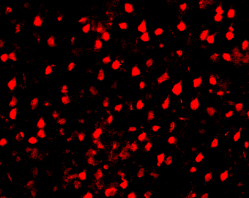

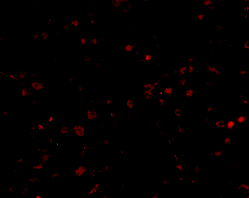

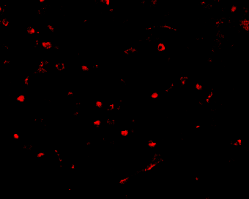

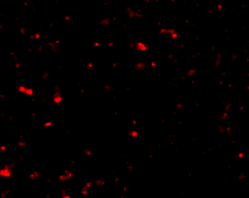

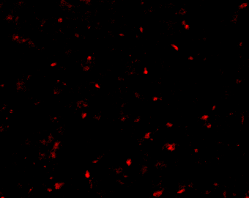

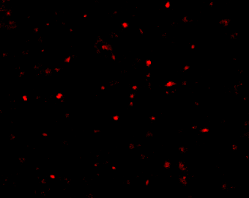

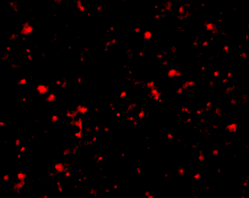

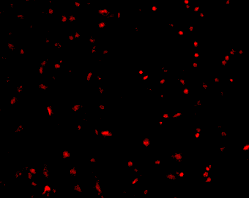

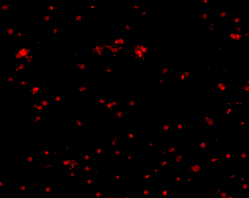

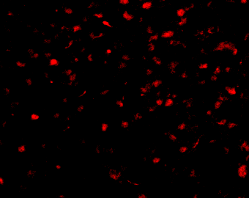

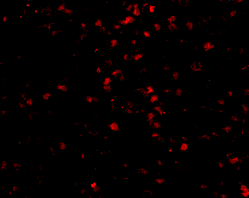

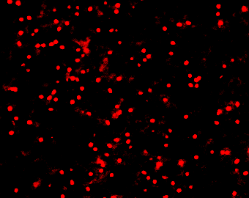

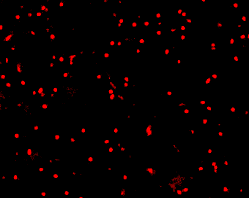

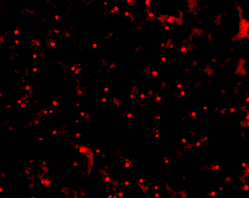

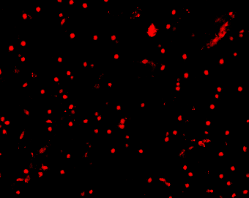

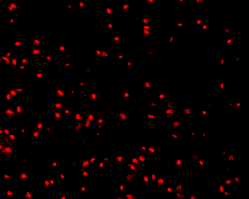
Total of four rows, from top to bottom, represent the Sham, MCAO, MSC, MSCHO-1 groups, and each group of 5 represents images
